# COVID-19 healthcare and social-related needs from the perspective of Spanish patients and healthcare providers: a qualitative analysis of responses to open-ended questions

**DOI:** 10.3389/fpubh.2023.1166317

**Published:** 2023-09-14

**Authors:** Andrea Duarte-Díaz, Mariana Aparicio Betancourt, Laura Seils, Carola Orrego, Lilisbeth Perestelo-Pérez, Jaime Barrio-Cortes, María Teresa Beca-Martínez, Carlos Jesús Bermejo-Caja, Ana Isabel González-González

**Affiliations:** ^1^Canary Islands Health Research Institute Foundation (FIISC), El Rosario, Spain; ^2^Network for Research on Chronicity, Primary Care, and Health Promotion (RICAPPS), Madrid, Spain; ^3^Avedis Donabedian Research Institute (FAD), Barcelona, Spain; ^4^Universitat Autònoma de Barcelona (UAB), Barcelona, Spain; ^5^Evaluation Unit (SESCS), Canary Islands Health Service (SCS), El Rosario, Spain; ^6^Instituto de Investigación Sanitaria Gregorio Marañón (IISGM), Madrid, Spain; ^7^Fundación Para la Investigación e Innovación Biosanitaria en Atención Primaria, Madrid, Spain; ^8^Faculty of Health Sciences, Universidad Camilo José Cela, Madrid, Spain; ^9^Unidad de Apoyo Técnico Dirección Técnica de Sistemas de Información, Gerencia Asistencial Atención Primaria, Servicio Madrileño de Salud, Madrid, Spain; ^10^Nursing Department, Universidad Autónoma de Madrid, Madrid, Spain; ^11^Unidad de Innovación y Proyectos Internacionales, Subdirección General de Investigación Sanitaria y Documentación, Dirección General Investigación y Docencia, Consejería de Sanidad, Madrid, Spain

**Keywords:** COVID-19, needs assessment, health services, social services, patients, health personnel, public health, Spain

## Abstract

**Introduction:**

Identifying stakeholders’ needs is crucial to informing decisions and policy development. This study aims to identify healthcare and social-related needs and effective strategies associated with COVID-19 from the first-person perspectives of patients and healthcare providers.

**Methods:**

Cross-sectional online survey design including qualitative open-ended questions, conducted in primary care and hospital settings across Spain, with 12 out of 19 regions represented. Adults aged 18 years and older, who (a) resided in Spain and had a history of COVID-19 or (b) worked as healthcare providers delivering direct or indirect care for people with COVID-19 in Spanish primary care or hospitals during 2020 were eligible to participate. Recruitment was conducted via social-media networks (Twitter, LinkedIn, and WhatsApp) and communication channels of key organizations including patient and professional associations and groups. A total of 182 people were invited to complete the surveys and 76 people completed the surveys (71% women), of which 33 were home-isolated patients, 14 were hospitalized patients, 16 were primary care professionals, and 13 were hospital care professionals.

**Results:**

A total of 327 needs and 86 effective strategies and positive aspects were identified across surveys and classified into the following overarching themes: (i) Accessibility, (ii) Basic needs, (iii) Clinical care, (iv) Person-and-family centered care, (v) Caring for the healthcare professional, (vi) Protocolization, information, health campaigns, and education, (vii) Resource availability, (viii) and Organizational needs/strategies.

**Discussion:**

Findings indicate the Spanish health and social care systems were generally unprepared to combat COVID-19. Implications for research, practice, and policy focus on integrating first-person perspectives as best practice to identify, prioritize and address needs to increase health and social care systems capacity and preparedness, as well as providing well-co-coordinated responses across government, healthcare, and non-government sectors to promote and protect the physical and mental health of all.

## 1. Introduction

The new coronavirus infectious disease (COVID-19) was first reported on December 2019, in Wuhan, China. The World Health Organization (WHO) declared the outbreak a pandemic shortly after, on March 11, 2020, because of the quick spread and severity of the disease (1, 2). The disease burden and mortality, the worsening of mental health and well-being, the delay of necessary and urgent care, and the acute economic paralysis caused by COVID-19, placed great pressure on society, public health and health systems (3–7).

The global health, social and economic crisis that ensued, exposed deficiencies in health systems and tested their public health preparedness and response capacity (8–10). The unprecedented nature of this novel coronavirus impacted the capacity of health systems to respond to the pandemic rapidly and effectively, with adequate personnel, equipment, medicines, and facilities (11–13). Limited or inadequate resources and ineffective public health policies, financial instability, and health information and communication challenges, impacted appropriate service delivery, particularly affecting the workforce, patients, and their family members (14).

The SARS-CoV-2 virus spread rapidly in Spain (15), one of Europe’s most affected countries, with 3,898,035 cases and 370,489 deaths notified as of July 7, 2021 (16). The first confirmed patient was diagnosed on January 31, 2020 (17) and by March 14, 2020, cases were confirmed in all Spanish regions. Spain declared a state of alarm on March 14, 2020, and introduced a hard nation-wide lockdown. By late-March 2020, hospitals were overwhelmed with COVID-19 patients, and intensive care units (ICU) in Catalunya, Comunidad de Madrid, Castilla-La Mancha, and Castilla y León were almost at full capacity, despite efforts to outnumber beds available. The burden of confirmed cases in ICUs was as high as in Italy and far above any other European countries (18). To combat the spread of the virus, on March 30, 2020 all non-essential workers were ordered to remain at home. On April 2, 2020, almost 1,000 people died due to COVID-19 in only 24 h, the highest number reached in one day (19). Health facilities in the worst affected regions were struggling, with ICUs still reaching capacity, an overall deficit in bed capacity, and insufficient number of health professionals, personal protective equipment (PPE) and ventilators. Catalunya, Comunidad de Madrid and other Spanish regions canceled non-emergency surgery and delayed non-COVID-19 urgent and follow-up care in primary care and hospitals because they were overwhelmed. COVID-19 telephone helplines collapsed in some regions. Healthcare professionals were redeployed, medical students were recruited, and retired healthcare workers were urged to return to work due to personnel shortages. In addition, the Spanish government took control of private health services, and military installations and hotels were used for public health purposes (15). The health crisis, along with public health safety measures introduced, such as social distancing, stay-at-home-orders, or school closures, severely impacted health professionals, as well as patients and their families (20).

In early-mid April, the number of patients in ICUs started to decrease in several Spanish regions including Comunidad de Madrid, Castilla-La Mancha, Principado de Asturias and Galicia (19). By mid-May 2020, the daily death count had fallen below 100 patients, and June 1, 2020 was the first day without deaths by COVID-19. Spanish health systems started to recover their normal functioning during June and the state of alarm regulations ended on June 21, 2020 (19).

The strict regulations enforced during the pandemic, however, affected the Spanish society and health system beyond the immediate impact of disease and deaths. This resulted in a decrease in the quality of health services and person-centered care, as well as extensive job losses and hardship, all of which caused a health, social, and economic critical situation (21, 22).

Given the profound multilevel impact of the pandemic, identifying and addressing COVID-19-related needs is warranted. This work is part of a larger COVID-19 needs project that seeks to explore healthcare and social-related needs during the care pathway of people with a history of COVID-19 to inform policy-making, clinical practice, and future research. The present study addresses the need elucidated by our previous work (14) to identify needs associated with COVID-19 from first-person perspectives. Here we aim to identify healthcare and social-related needs and effective strategies associated with COVID-19 from the perspective of patients and the healthcare providers involved in their care, in primary and hospital care settings in Spain.

## 2. Methods

### 2.1. Design

A cross-sectional online survey design including qualitative open-ended questions was used to identify COVID-19 healthcare and social-related needs and effective strategies in Spain from the perspective of patients and healthcare providers.

### 2.2. Participants

Adults aged 18 years and older who (a) resided in Spain and had a history of COVID-19 and required home isolation or hospital admission, and/or (b) worked as healthcare providers, delivering direct or indirect care for people with COVID-19, in Spanish primary care or hospitals during 2020, were eligible to participate in this study. Participants were excluded if they were unable or refused to provide informed consent.

### 2.3. Instrument

Four *ad hoc* surveys using open-ended questions were designed by the research team, targeting (1) people with a history of COVID-19 isolated at home, (2) people with a history of COVID-19 who were hospitalized, (3) primary care professionals involved in the care of people with COVID-19, and (4) hospital care professionals involved in the care of people with COVID-19. Surveys were designed based on the healthcare and social-related needs associated with COVID-19 identified after the literature review conducted by our research team (14), a patient perspective, and the expert opinion of the research team, a multidisciplinary team representing the following disciplines: psychology, speech-language pathology, physical therapy, nursing, medicine, neuroscience, quality improvement, patient safety, and healthcare administration. Each survey consisted of three main sections: Informed consent, sociodemographic questions, and a set of four to six qualitative open-ended questions allowing participants to provide detailed responses related to difficulties, areas for improvement, or unmet needs throughout the patient care pathway, as well as an additional question related to effective strategies and positive aspects. Professionals’ surveys included two additional questions related to their work and personal environment. Stages and categories captured by the open-ended questions varied by survey and are listed in Table 1. Prior to distribution, the surveys were iteratively reviewed by the research team. Surveys were developed with SurveyMonkey^®^ and distributed via email. Surveys were designed so participants had to answer each question in a section before proceeding to the next section to ensure there was no missing data from skipped questions. Each survey lasted approximately 10–20 min (Supplementary material S1 provides the Spanish to English translated survey questionnaires).

**Table 1:** Stages and categories captured by the open-ended questions in each of the four surveys.

	Patients	Professionals
Primary care	Contact/ onset of symptoms Diagnosis Visits Telephone monitoring and home isolation Primary care discharge Effective strategies	Contact/ onset of patient symptoms Diagnosis Visits Telephone monitoring and home isolation Primary care discharge Work environment Personal environment Effective strategies
Hospital care	Contact/ onset of symptoms Diagnosis Emergency care Hospitalization Intensive Care Unit care Hospital discharge Effective strategies	Emergency care Hospitalization Intensive Care Unit care Hospital discharge Work environment Personal environment Effective strategies

### 2.4. Data collection

The surveys were open from March 23, 2021, to July 2, 2021. They were launched during the fourth COVID-19 wave in Spain (March 15 – June 19) to gather the perspectives of patients and professionals regarding current and past healthcare and social-related needs and effective strategies associated with the pandemic. We attempted to obtain representation across Spanish regions, from patients across the age span and in need of different clinical services (e.g., ICU admission), and from multiple professional disciplines (medical doctors, nurses, and if possible one other discipline). Regions were divided into four groups, of four to six regions each, based on the percentage of COVID-19 cases in Spain according to the Institute of Health Carlos III (ISCIII) COVID-19 report as of March 17, 2021 (23): 0–1% (Principado de Asturias, Canarias, Cantabria, Ceuta, La Rioja, Melilla), 2–3% (Aragón, Islas Baleares, Extremadura, Comunidad Foral de Navarra), 4–6% (Castilla y León, Castilla-La Mancha, Galicia, Región de Murcia, País Vasco), and greater than 6% (Range: 13–18%) (Andalucía, Catalunya, Comunidad de Madrid, Comunidad Valenciana). We aimed to obtain responses from at least two region groups per survey. Surveys were closed based on thematic saturation, representation of different sociodemographic characteristics (region group, age, professional occupation, and ICU admission), and time elapsed.

Participants first completed a patient or professional registration form using Google^®^ Forms, after which the research team verified whether participants met inclusion criteria and sent a follow-up email with the respective survey link(s), if appropriate. One reminder email was sent at least one week after the initial email. Eligible participants then had the opportunity to access the consent form and information sheet describing the research and the procedures for ensuring data confidentiality via the survey link(s). Once informed consent was provided, participants were allowed to complete the remaining sections of the survey. The research team provided technical assistance to complete the surveys when requested.

Recruitment focused on disseminating study information together with the registration form links via social networks (Twitter, LinkedIn, WhatsApp), as well as via the various communication channels of key organizations including the researchers’ home institutions (Avedis Donabedian Foundation, FAD; Evaluation and Planning Service of the Canary Islands Health Service, SESCS; Participa y Decide sobre tu Salud, PyDeSalud; Servicio Madrileño de Salud, SERMAS), and patient and professional associations and groups (e.g., Andalusian platform of patients affected by long-COVID; Spanish Society of Family and Community Medicine, semFYC). We relied on convenience and snowballing methods; while we exploited personal and professional contacts, we also sent cold emails as well as tagged influencers and groups to personal and institutional posts on Twitter and LinkedIn.

### 2.5. Data analysis

The data sets were downloaded from SurveyMonkey^®^ into Microsoft^®^ Excel. Responses with 100% of missing qualitative data were excluded from the analysis. Qualitative data were exported to Microsoft^®^ Word and thematically analyzed by two reviewers using a mixed inductive-deductive essentialist or realist approach to identify themes at a semantic level. Thematic analysis is a qualitative analytic method “for identifying, analyzing, and reporting patterns (themes) within data” (24).

Reviewers first familiarized themselves with the data, by actively reading, re-reading, and discussing the data. Subsequently, a first reviewer (ADD) generated initial codes, and combined the codes into identified healthcare and social-related needs and effective strategies associated with COVID-19. Codes, needs, and strategies were then reviewed in-depth by a second reviewer (MAB). During a third round of revision, reviewers discussed and reached consensus to develop a final list of needs and strategies by subgroup. For each survey, needs were divided by the pre-established categories in the care pathway and an additional transversal category was created during the consensus round. The data analysis process and the final lists of needs and strategies were shared with the research team and further refined. Additionally, to ensure comprehensibility, each final list of patient needs (isolated at home or requiring hospital admission) was reviewed by a COVID-19 patient. As a result of the feedback, minor wording modifications were made to improve clarity. These lists of needs are the basis for a set of follow-up quantitative surveys developed to prioritize and obtain consensus over the identified needs.

For the purposes of this work, we completed four additional coding passes to classify the identified needs across surveys into overarching themes and subthemes. A first approach to classifying the needs into themes and subthemes was completed by a third reviewer (CBJ), and discussed with the initial two reviewers (ADD, MAB). ADD or MAB subsequently conducted an in-depth coding pass of the identified needs, after which coding was validated by either MAB or ADD during the third coding pass. During the fourth and final round of revision, the initial two reviewers discussed and reached consensus. The codebook was further developed, refined, and discussed throughout the coding process and needs were recoded as needed. Identified needs were coded in more than one subtheme when deemed necessary. Similarly, effective strategies were coded (MAB) into the aforementioned themes and subthemes and validated by a second reviewer (ADD). Disagreements were resolved by consensus.

### 2.6. Patient and public involvement

One patient with a history of COVID-19 isolated at home was interviewed over the phone to describe her journey of care and associated healthcare and social-related needs, to inform the design of the surveys. Two additional patients were involved in reviewing the identified patient needs. Articles and reports published as a result of the larger COVID-19 needs research project will be shared with the patients involved and they will be encouraged to disseminate the results widely.

## 3. Results

### 3.1. Study population characteristics

Of the 196 registration forms received (103 patient forms and 93 professional forms), twelve did not meet inclusion criteria: six people who responded the patient registration form had no history of COVID-19, and six professionals who responded the professional registration form worked outside of primary or hospital care (e.g., nursing home, psychiatric center). Accordingly, 184 people were invited to complete the survey(s) via email, of which two email addresses were incorrect. Of the 105 survey responses received, 29 were excluded due to missing 100% of the qualitative data. A total of 76 people completed the surveys and were included in the analysis: 33 home-isolated patients with COVID-19, 14 hospitalized patients with COVID-19, 16 primary care professionals, and 13 hospital care professionals.

**Figure 1:**
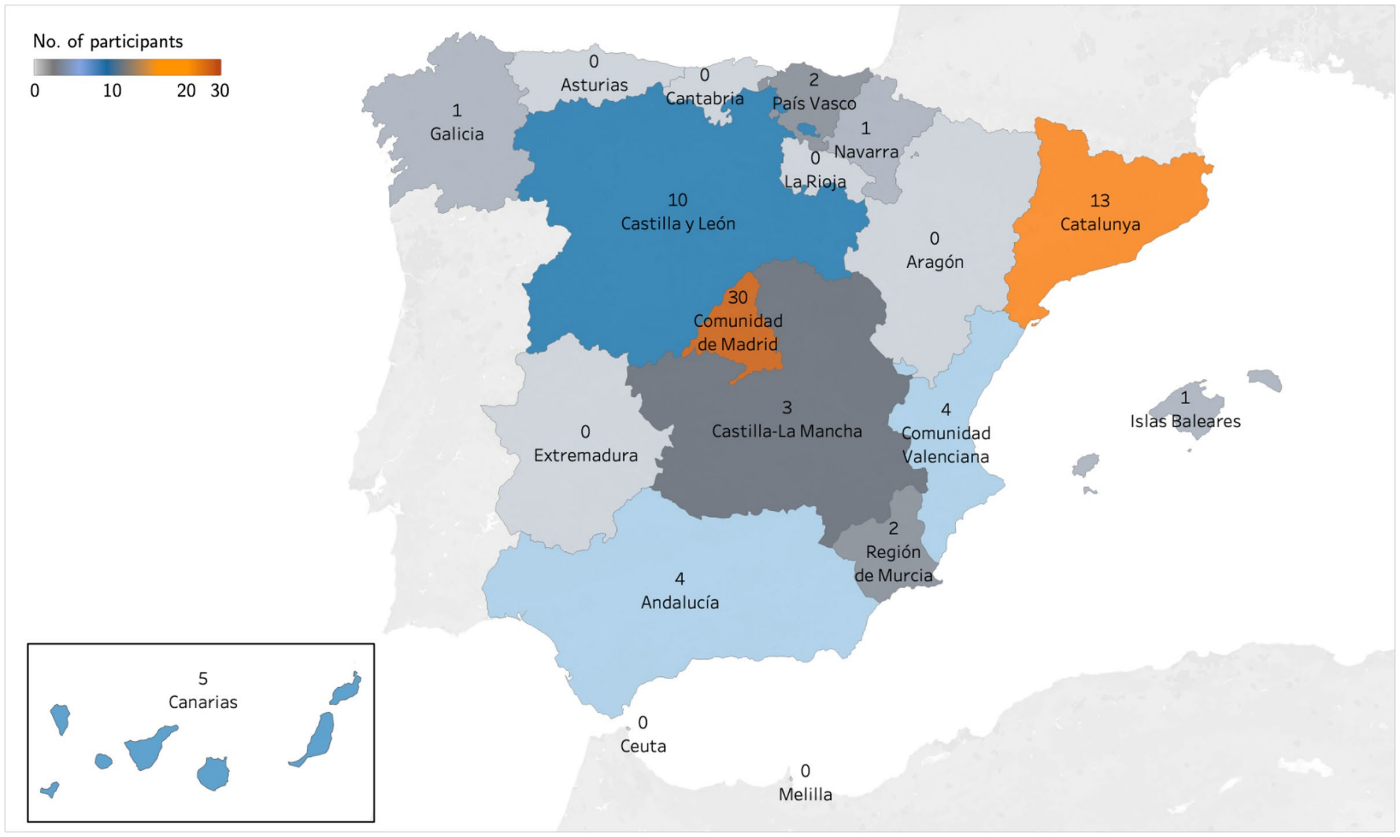
Distribution of participants by Spanish region.

Overall, we obtained representation from all four region groups, with 12 of the 19 Spanish regions represented. Patients’ subgroups had representation from 12 of 19 Spanish regions and all four region groups, while professionals’ subgroups had representation from 7 of 19 Spanish regions and 3 of 4 region groups. The most represented region was Comunidad de Madrid (*n* = 30, 15 patients and 15 professionals; 39.47%), followed by Catalunya (*n* = 13, 9 patients and 4 professionals; 17.11%) and Castilla y León (*n* = 10, 9 patients and 1 professional; 13.16%). Figure 1 summarizes the distribution of participants by Spanish region.

The mean age of the whole sample was 46.73 years (SD = 12.15) and 71.05% were women. Most patients (*n* = 39; 82.98%), including those isolated at home and those hospitalized, had an associate’s degree or higher. The majority of professionals were physicians (*n* = 21; 72.41%) or nurses (*n* = 7; 24.14%), with only one responder employed in a different discipline (i.e., psychology). Patients who participated in the surveys were diagnosed with COVID-19 or first presented COVID-19 symptoms from March 7, 2020 to April 11, 2021. Less than half of the COVID-19 patients (*n* = 19; 40.43%) reported fully recovering, with 2 to 270 days of symptom duration (*M* = 39.95, SD = 61.25). On the other hand, 59.57% (*n* = 28) of patients had not fully recovered from COVID-19 symptoms when they responded to the survey between March and July, 2021, and still had persistent symptoms including changes in smell and taste perception, muscle and joint pain, fatigue, dyspnea and cognitive impairment. Table 2 provides a description of the sociodemographic characteristics by study subgroup.

**Table 2:** Sociodemographic characteristics by study subgroup.

	Patients isolated at home	Patients requiring hospital admission	Primary care professionals	Hospital care professionals
n/N (%)	33/76 (18.42)	14/76 (43.42)	16/76 (21.05)	13/76 (17.11)
Mean age (Standard deviation)	46.73 (12.15)	48.64 (10.80)	52.13 (11.83)	37.85 (9.78)
Gender^a^ (*n*, %)				
Women	27 (81.82)	7 (50)	10 (62.50)	10 (76.92)
Men	6 (18.18)	7 (50)	6 (37.50)	3 (23.08)
Educational level (*n*, %)				
Primary education	1 (3.03)	0 (0)	–	–
Secondary education	3 (9.09)	4 (28.57)		
Associate’s degree or higher	29 (87.88)	10 (71.43)		
Profession (*n*, %)				
Physician	–	–	10 (62.50)	11 (84.62)
Nurse			6 (37.50)	1 (7.69)
Psychologist			0 (0)	1 (7.69)
Spanish regions (*n*, %)				
0–1%	1 (3.03)	0 (0)	2 (12.5)	2 (12.5)
Canarias	1 (3.03)	0 (0)	2 (12.5)	2 (12.5)
2–3%	1 (3.03)	1 (7.14)	0 (0)	0 (0)
Islas Baleares	1 (3.03)	0 (0)	0 (0)	0 (0)
Comunidad Foral de Navarra	0 (0)	1 (7.14)	0 (0)	0 (0)
4–6%	6 (18.18)	9 (64.28)	0 (0)	3 (23.07)
Castilla y León	1 (3.03)	8 (57.14)	0 (0)	1 (7.69)
Castilla-La Mancha	1 (3.03)	0 (0)	0 (0)	2 (15.38)
Galicia	0 (0)	1 (7.14)	0 (0)	0 (0)
Región de Murcia	2 (6.06)	0 (0)	0 (0)	0 (0)
País Vasco	2 (6.06)	0 (0)	0 (0)	0 (0)
>6%	25 (75.76)	4 (28.58)	14 (87.5)	8 (61.53)
Andalucía	3 (9.09)	0 (0)	1 (6.25)	0 (0)
Catalunya	7 (21.21)	2 (14.29)	4 (25)	0 (0)
Comunidad de Madrid	13 (39.39)	2 (14.29)	9 (56.25)	6 (46.15)
Comunidad Valenciana	2 (6.06)	0 (0)	0 (0)	2 (15.38)
Total number of regions represented	10/19	5/19	4/19	5/19
Total number of region groups represented	4/4	3/4	2/4	3/4

^a^Participants were provided the option to indicate they identify as woman, man, or nonbinary in alignment with inclusive language guidelines [e.g., Cameron and Stinson (25)].

### 3.2. COVID-19 healthcare and social-related needs

We focused on the perceived healthcare and social COVID-19-related needs of patients and professionals in primary and hospital care in Spain. Thematic data analysis in this study led to the identification of 81 needs reported by home-isolated patients, 62 needs reported by hospitalized patients, 94 needs reported by primary care professionals, and 90 needs reported by hospital care professionals, for a total of 327 needs across surveys. Identified needs were classified across eight themes and 32 subthemes (Table 3; Supplementary material S2 provides the Spanish to English translated list of needs).

**Table 3:** Key themes and subthemes of COVID-19 healthcare and social-related needs.

Theme	*n* (%)^a^	Subgroup	Illustrative response
Accessibility needs	58 (17.06)	All	Patients isolated at home, Participant 13: (Diagnostic testing) *“The data of my negative PCR were not released until 72 h later and, on top of that, they did it with an erroneous result. I was discharged and allowed to return to work, as they trusted the negative result that I communicated by phone…”*
Early symptoms	5	HI, HA, HC	
Diagnostic testing	15	All	
Emergency care	4	HI, PC	
Treatment & rehabilitation services	7	HI, HA	
Non-COVID-19 care	4	All	
Occupational health services	4	HI	
Multimedia communication	10	All	
Efficient and continuous access	9	HI, HA, PC	
Basic needs	17 (5)	All	Patients requiring hospital admission, Participant 5: (Essential supplies and resources, Healing environment) *“You cannot have three sick people in a room, with almost no ventilation, a small bathroom for the three of us, people who did not even have a shower to properly wash themselves.”*
Essential supplies and resources	9	All	
Work conditions & financial assistance	3	HI	
Healing environment	5	HA	
Clinical care needs	30 (8.82)	All	Patients isolated at home, Participant 28: (High-quality care) *“… Everything was focused on the most severe cases. Those of us who could not receive care in hospitals at the beginning of the epidemic have been ignored. We do not even register as infected, as we did not take a PCR test. My only treatment was paracetamol and an antibiotic a month after getting sick. All by phone and with different doctors. Without even an X-ray. Also note the lack of empathy in dealing with the sick of many health care providers.”*
Improving patient experience	9	All	
Patient monitoring & follow-up care	21	All	
Person-and-family centered care needs	47 (13.82)	All	Patients requiring hospital admission, Participant 4: (Culture of respect) *“Incomprehension on the part of my primary care physician. He has always considered that all my symptomatology was derived from work stress and that I was somatizing. In fact, I was even admitted to the psychiatric unit.”*
Culture of respect	12	All	
Psychological health	13	All	
Individualized care	8	HI, HA	
Family involvement and communication	14	All	
Caring for the healthcare professional needs	41 (12.06)	Pt: HA, Prof: PC, HC	Primary care professionals, Participant 4, Physician: (Occupational health and safety) *“In the first months there has been a lack of all kinds of protective material for both in-center and home care, resorting to everything we could think of (garbage bags, raincoats).”*
Support from leaders and managers	5	PC, HC	
Occupational health and safety	13	HA, PC, HC	
Psychological health	12	PC, HC	
Social support & work-life balance	11	PC, HC	
Protocolization, information, health campaigns, & education needs	67 (19.71)	All	Primary care professionals, Participant 15, Nurse: (Protocolization) *“Frequent organizational changes [.] Having to stop performing functions proper to my profession [.] Frequent changes in the protocols of action.”*
Protocolization	18	All	
Information	18	All	
Public health disease prevention measures	12	All	
Education & training	19	All	
Resource availability needs	45 (13.24)	All	Hospital care professionals, Participant 12, Physician: (Human resources, General or other material resources) *“The doctors’ work overload, most of the time, does not allow us to contact family members by telephone to inform them of the situation (through a switchboard that is also collapsed).”*
Human resources	21	All	
Infrastructure (digital & non-digital)	12	HA, PC, HC	
Transportation	5	HA, PC, HC	
General or other material resources	7	HA, PC, HC	
Organizational needs	35 (10.29)	All	Hospital care professionals, Participant 1, Nurse: (Coordination and communication, Organizational changes) *“I was afraid of making mistakes that could affect the patient. After four years without being a practitioner, returning to work under these conditions created some anxiety. … My responsibilities as patient safety nurse coordinator have been suspended, we have not held group meetings again, there are no ‘discussions’ about implementing measures to improve safety. On a daily basis, our actions jeopardize patient safety, but during the first wave, too many adverse events have happened. Many, many communication problems. They were already present before and now between the mask, the isolation measures… There could be an improvement opportunity if managers were aware of the errors that have been made and were interested in analyzing them and implementing measures so that they do not happen again, but I have not seen that attitude.”*
Coordination and communication	14	HI, PC, HC	
Job security, fair pay, and workers’ rights	5	HI, PC, HC	
Organizational changes	16	All	

Illustrative quotes were translated from Spanish to English. Pt. represents patients; HI represents patients who required home isolation; HA represents patients who required hospital admission; Prof. represents professionals; PC represents primary care professionals; HC represents hospital care professionals; PCR, represents polymerase chain reaction. ^a^*n* represents the number of needs identified across surveys (*N* = 340). Note that the total number of needs reported in this table reflects multiple counting as *(i)* 13 needs were coded in more than one subtheme, and *(ii)* similar or the same needs may have been reported in more than one survey.

#### 3.2.1. Accessibility needs

Representatives from the four population subgroups reported 58 accessibility needs (17.06%). As soon as the first symptoms appear, citizens must be able to rapidly contact health services and providers to aid in early detection via multiple communication media, and obtain quick access to accurate diagnostic tests and results close to their homes or at-home. Patients emphasized access to diagnostic tests and results should also be extended to close contacts. Additionally, patients stressed the importance of being able to contact emergency services when needed, including on weekends and holidays, and via a variety of channels. Whereas home-isolated patients underscored the importance of immediate access to urgent medical transport, hospital professionals requested prompt attention from emergency services for transporting patients to the hospital in the event of an emergency. Once a diagnosis has been confirmed, patients indicated the need to access an adequate treatment plan, access the providers in charge of their care, and rehabilitation services, particularly in the event of long-term COVID-19 complications. Patients isolated at home also emphasized the importance of having easy and quick access to Occupational Health Services (OHS) after a diagnosis in order to request sick leave, and similarly, primary care professionals suggested online access to OHS could help avoid displacing home-isolated patients to healthcare centers. Moreover, addressing non-COVID-19 healthcare needs of COVID-19 and/or non-COVID-19 patients also emerged as a need from the perspective of both patients and professionals (e.g., chronic diseases, palliative care, care for people with mobility impairments). Overall, all participants highlighted the need for multiple communication media, based on patients’ needs and preferences, such as videoconferences, home visits, in-person, or phone calls, in order to timely access health services. Finally, it was noted by both patients and primary care professionals that access to care must be efficient and continuous. To make this possible, there is a need to reduce waiting times in primary care, emergency care, and with specialists in hospital settings, improve communication, provide timely in-person care and care during extended hours, prioritize cases, have sufficient personnel, and provide administrative solutions to exceptional cases.

#### 3.2.2. Basic needs

Seventeen (5%) basic needs were identified by participants from all subgroups. Needs related to the provision of essential supplies and resources were reported by all participants. While hospital care professionals indicated hospitalized patients required access to their personal belongings, hospitalized patients requested adequate food service and bathroom facilities, as well as assistance with personal hygiene. In turn, primary care professionals and home-isolated patients, reported patients isolated at home required access to facilities to safely isolate themselves from other household members, as well as support during isolation such as caring for dependents or with domestic tasks (e.g., home-delivery of food and medicine, cleaning, etc.). Additionally, patients who were isolated at home emphasized the importance of flexible working conditions, including remote work, and financial support to deal with the economic consequences of isolation. Moreover, a healing hospital environment emerged as a subtheme for hospitalized patients, who indicated the need to reduce environmental stressors such as noise and poor air quality, to minimize patient movement, and the need for rapid access to a hospital room.

#### 3.2.3. Clinical care needs

Clinical care needs (*n* = 30, 8.82%) were identified by all subgroups. To ensure patients receive high-quality care and to improve the patient experience, first and foremost, improvements in the diagnosis phase are required, including increased diagnostic test reliability, and implementation of a wide testing strategy that includes testing asymptomatic persons who are close contacts. Next, patients must have access to effective treatment plans from the onset of symptoms, even if they are in home isolation. Improvements in information provision for patients and their caregivers on the treatment and discharge plans are also needed; written, simple, and easy to understand information is key. Moreover, rigorous and continuous patient monitoring for patients in home isolation with or without a confirmed diagnosis and hospitalized patients, as well as follow-up care to ensure patient compliance, symptom control, and continuity of care are also required, as reported by all subgroups.

#### 3.2.4. Person-and-family centered care needs

Forty-seven (13.82%) needs identified by all subgroups were classified in the Person-and-family centered care theme. All subgroups placed emphasis on building a culture of respect characterized by empathy, respect, affectionate and effective communication, active listening, shared decision-making, and trusting relationships. Mental health and emotional support for patients and their loved ones during home isolation, hospitalization, ICU admission, and post-hospital discharge emerged as another subtheme stressed by all subgroups. Primary care professionals also expressed the need to embrace health from a social perspective that includes accessible public health initiatives to promote mental health. Additionally, patients noted the importance of individualized care, particularly in relation to communicating with the same professional during their care journey, treatment and discharge decisions adapted to their specific circumstances, adapting communication to patients’ needs and preferences, and support scheduling medical appointments. Finally, all subgroups emphasized the need to keep loved ones involved and in continuous communication with patients and professionals throughout the patient care journey (e.g., verbal and written information sharing, communicating via phone or videoconference, hospital visits). In-person visits were particularly stressed for children, older adults, disabled people, and terminally ill people by professionals.

#### 3.2.5. Caring for the healthcare professional needs

Forty-one (12.06%) needs, primarily reported by primary and hospital care professionals, were concerned with caring for the healthcare professional. Real support from leaders and managers and recognition of the work of professionals were seen as crucial. To this end, professionals requested face-to-face visits by management, good coordination, adaptive leaders, and comprehension. Additionally, occupational health and safety was reported as a main priority for professionals. Key elements identified included the provision and proper use of adequate PPE that does not hinder visibility and comfort, which was also identified as a need by hospitalized patients; implementation of organizational preventive actions and strategies and improving adherence to such measures; reduced work shifts and a reasonable workload allowing for time to rest during and outside the work environment; working within healthcare providers’ usual scope of practice or receiving appropriate redeployment training; and the option to carry out low-risk work for professionals particularly vulnerable to COVID-19. Caring for professionals’ psychological health emerged as another subtheme. Further, primary and hospital care professionals emphasized the need for continuous proactive evaluation of physical and psychological health and tangible actions to address needs, as well as support to manage stress, anxiety, fear of infection, continuous isolation, and the COVID-19 crisis in general for themselves and their families. Finally, professionals noted the need for work-life balance, and for organizations to provide social support to care for dependents and when infected with COVID-19 (lodging, food, financial assistance, etc.). The need for social connection and public health measures to reduce stigmatization also emerged.

#### 3.2.6. Protocolization, information, health campaigns, and education needs

One fifth of needs (*n* = 67, 19.71%) recognized the need for protocolization, information, public health disease prevention measures, and education and training. Patients and professionals required clear, accessible, effective, and adequately implemented protocols and decision algorithms throughout the journey of care. Although all participants stressed the importance for the existence and continuous update of protocols, professionals emphasized the need to avoid unnecessary changes, for managers to highlight the relevant changes, as well as to maintain certain degree of flexibility (e.g., based on clinical judgment, shared-decision making). Additionally, clear action protocols for specific circumstances and vulnerable populations, such as caring for a nursing infant when the caregiver has COVID-19 or for the protection of vulnerable people when reentering the workforce, were required by home-isolated patients. Information about how to proceed in specific situations and more broadly, consistent, concise, up-to-date, and clear information about COVID-19 provided by healthcare professionals, public health, and communication media was needed for patients, their relatives, and citizens (e.g., early detection of symptoms, when and how to isolate, infection control, antibody generation and possibility of reinfection, legal information related to medical discharge and work reinstatement). Although informational needs were reported by all subgroups, they were mainly stressed by patients. Moreover, public health measures using multiple modes of communication (e.g., verbal and written instructions, video tutorials, etc.) to prevent the spread of the disease, avoid the spread of fear and misinformation, and reduce stigmatization of people infected with COVID-19 were also needed. Finally, all subgroups identified gaps in the education and training of professionals related to early identification of symptoms and diagnosis, patient monitoring and follow-up care, long COVID, redeployment, correct donning and doffing PPE in different settings, telehealth, counseling and communication with patients and their relatives, resident training, and knowledge and practices concerning COVID-19 more broadly.

#### 3.2.7. Resource availability needs

Forty-five (13.24%) needs reported by all subgroups were about availability of human and material resources to meet the rise in demand for care and avoid the saturation of health systems. Participants in all subgroups identified the need to hire additional qualified staff including healthcare providers (e.g., to maintain reasonable workloads and work shifts, avoid redeployments, provide non-COVID-19 care, improve triage and diagnostic testing for early identification and separation of suspected COVID-19 patients, provide support with activities of daily living, improve patient monitoring of COVID-19 and non-COVID-19 cases, improve communication with relatives), administrative personnel (e.g., to improve contact tracing, appointment management), IT staff, support personnel to deliver food and other essential resources to patients isolated at home, and mental health professionals for patients, family members, and citizens. Subthemes related to material resources availability included transportation, digital and nondigital infrastructure, and general or other material resources. Transportation resources were needed for both patients (e.g., ambulances, after hospital discharge) and professionals (e.g., during isolation, to health centers, for home-care services). The need to improve digital technologies and for the provision of sufficient and adequate spaces (to preserve privacy, separate cases, non-COVID-19 treatment, increase bed and ICU capacity) were also noted by professionals and hospitalized patients. Other material resources required reported mainly by professionals, included reagents, diagnostic tests, access to x-rays and ultrasounds in primary care, and face masks for patients, professionals and citizens.

#### 3.2.8. Organizational needs

Thirty-five (10.29%) needs reported by all subgroups focused on coordination and communication, work conditions, and organizational changes. Home-isolated patients and primary and hospital care professionals highlighted the need for improved intra- and inter-professional coordination and communication within departments, within health centers, between health services, and/or between health authorities and health providers, to: improve early detection and disease prevention; improve patient monitoring, follow-up care, and patient care more broadly; improve work conditions; and promote knowledge exchange between professionals. Professionals and home-isolated patients also reported needs related to job security, fair pay, sick leave, and workers’ rights including “maintenance of work conditions or consensual changes” (e.g., place of work, schedules, shifts, tasks). Organizational changes to better address the COVID-19 crisis and future global threats were also identified by all subgroups. Patients and professionals reported the need to improve contact tracing, and to reduce or prevent medical errors (e.g., related to diagnosis) by improving organization. Additionally, professionals also reported the need to avoid pauses or delays in non-COVID-19 care; improve patient monitoring; promote patient safety; reduce the need to improvise and the saturation of health systems (by, e.g., having primary care provide a treatment plan, increase automation of tasks); improve quality control measures and quality improvement more broadly by, for example establishing a systematic and continuous evaluation of service needs and implementing the necessary actions to address them; develop adaptive capacity; and improve decision-making processes to involve frontline professionals and organizational transparency.

### 3.3. COVID-19 healthcare and social-related effective strategies and positive aspects

Although the present study primarily focused on identifying needs, effective strategies and positive aspects related to the COVID-19 patient care pathway were also reported. A total of 86 effective strategies and positive aspects were reported across surveys: 22 by home-isolated patients, 9 by hospitalized patients, 35 by primary care professionals, and 20 by hospital care professionals. Effective strategies and positive aspects were classified across seven themes and 21 subthemes (all of the aforementioned themes except the Basic needs theme) (Table 4; Supplementary material S3 provides the Spanish to English translated list of effective strategies and positive aspects).

**Table 4:** Key themes and subthemes of COVID-19 healthcare and social-related effective strategies and positive aspects.

Theme	*n* (%)^a^	Subgroup	Illustrative response
Accessibility	8 (9.30)	Pt: HI, Prof: PC	Primary care professionals, Participant 16, Nurse: (Diagnostic testing) *“Accessible AIDTs [active infection diagnostic tests] for early detection.”*
Early symptoms	1	HI	
Diagnostic testing	4	HI, PC	
Treatment & rehabilitation services	2	HI	
Efficient and continuous access	1	HI	
Clinical care	10 (11.63)	Pt: HI, HA	Patients isolated at home, Participant 8: (Patient monitoring & follow-up care) *“[I received] good [patient] care… I received periodic follow-up care from my doctor with recommendations for treatment based on my progress and once [I recovered] they discharged me.”*
Improving patient experience	7	HI, HA	
Patient monitoring & follow-up care	3	HI, HA	
Person-and-family centered care	8 (9.30)	All	Hospital care professionals, Participant 6, Resident Psychologist: (Psychological health) *“From my service, Mental Health, a liaison program was created to help health workers, patients, and family members affected by COVID.”*
Culture of respect	2	HI, HA	
Psychological health	4	HI, PC, HC	
Individualized care	2	HI	
Caring for the healthcare professional	9 (10.47)	Prof: PC, HC	Hospital care professionals, Participant 11, Resident Physician: (Occupational health and safety) *“In some services [the teams] were divided into morning and afternoon shift[s] and I think that this helps a lot to unload the work of the 24-h shifts without neglecting the service, even though they complained that they did not have the financial support of the 24-h shifts.”*
Occupational health and safety	6	PC, HC	
Psychological health	1	HC	
Social support & work-life balance	2	PC, HC	
Protocolization, information, health campaigns, & education	24 (27.91)	All	Primary care professionals, Participant 16, Nurse: (Public health disease prevention measures) *“Mass vaccination of the population, starting with the most vulnerable people.”*
Protocolization	2	PC, HC	
Information	4	HI, PC	
Public health disease prevention measures	15	HI, PC, HC	
Education & training	3	HI, HA	
Resource availability	6 (6.98)	Prof: PC, HC	Hospital care professionals, Participant 2, Physician: [Infrastructure (digital & non-digital)] *“Increas[ing] the number of ICU and Internal Medicine beds with medical tents so as not to stop the rest of the care activity.”*
Human resources	2	PC, HC	
Infrastructure (digital & non-digital)	3	PC, HC	
General or other material resources	1	HC	
Organizational strategies	21 (24.42)	Pt: HI, Prof: PC, HC	Primary care professionals, Participant 6, Resident Physician: (Coordination and communication) *“Distribution of roles & responsibilities among professionals at the center.”*
Coordination and communication	9	HI, PC, HC	
Organizational changes	12	PC, HC	

Illustrative quotes were translated from Spanish to English. Pt. represents patients; HI represents patients who required home isolation; HA represents patients who required hospital admission; Prof. represents professionals; PC represents primary care professionals; HC represents hospital care professionals.^a^*n* represents the number of effective strategies and positive aspects reported across surveys (*N* = 86). Note that the number of effective strategies and positive aspects across surveys may reflect multiple counting, as similar or the same effective strategies or positive aspects may have been reported in more than one survey; within each survey, the number of effective strategies and positive aspects are distinct, thus not reflecting multiple counting.

Thirty-one (36.04%) effective strategies and positive aspects were reported by patients. These focused on *Accessibility*, including access to health services/providers to aid in early detection, access to rapid diagnostic testing, access to treatment and rehabilitation services to address physical and cognitive symptoms, and efficient and continuous access to healthcare providers via phone; *Clinical care*, including improving patient experience and satisfaction, effective and prompt treatment, and rigorous patient monitoring and follow-up care; *Person-and-family centered care*, including providing individualized and empathetic care, and establishing community support networks; *Protocolization, information, health campaigns, and education* by investing in research, keeping citizens well-informed, continuous training of healthcare personnel on COVID-19 including long-COVID, and public health disease prevention measures such as contact tracing and quick isolation of confirmed or suspected cases; and *Organizational strategies*, including improving coordination between health services and effective referrals.

On the other hand, fifty-five (63.95%) effective strategies and positive aspects were reported by professionals. Strategies focused on *Accessibility* to diagnostic testing for early detection; *Person-and-family centered care*, including establishing community support networks and mental health programs for patients and their relatives; *Caring for the healthcare professional* by ensuring sufficient and appropriate use of PPE, enforcing stringent hygiene, ventilation, and other biosecurity measures, reorganizing work shifts, developing mental health programs for professionals and their relatives, and establishing community support; *Protocolization, information health campaigns, and education*, including developing and adapting protocols as needed, providing up-to-date information for patients and professionals via multiple channels, learning of and sharing local care networks/resources with patients, and public health disease prevention measures such as performing diagnostic tests outdoors, limiting the maximum occupancy capacity, rapid isolation of confirmed or suspected cases, public awareness campaigns, mass screening, diagnostic testing, and vaccination, effective and efficient contact tracing, and enforcing biosecurity measures such as mandatory use of masks and social distancing; *Resource availability*, including hiring additional personnel, adapting outdoor and indoor spaces and ensuring the availability of other material resources to meet the demand for care; and *Organizational strategies*, including improving collaboration within teams, centers, health services, and neighborhoods, effective referrals, redistributing roles and responsibilities, permitting changes in appointments, adaptive capacity of the team and the organization, timely implementation of changes, increasing telephone appointments, implementing effective screening and diagnostic protocols, and establishing closed circuits for COVID-19 patients and independent working groups for professionals.

## 4. Discussion

As part of a larger mixed methods project seeking to explore COVID-19-related healthcare and social needs, the present study explored the needs and effective strategies associated with COVID-19 from the perspective of patients and healthcare providers in two different healthcare settings: Primary care and hospitals. A total of 327 needs and 86 effective strategies and positive aspects were identified based on first-person perspectives and classified into the following eight overarching themes: (i) Accessibility, (ii) Basic needs, (iii) Clinical care, (iv) Person-and-family-centered care, (v) Caring for the healthcare professional, (vi) Protocolization, information, health campaigns, and education, (vii) Resource availability, (viii) and Organizational needs/strategies. Of interest, most of the effective strategies reported by the participants overlapped with the identified needs, highlighting the importance of prioritizing and addressing the needs identified (the association between identified needs and effective strategies is further discussed in Section 4.1).

Overall, health and social systems of care were generally unprepared for a crisis like the COVID-19 pandemic (15, 26); this was also the case for Spanish systems of care. Our findings strongly align with the Health Foundation’s dimensions of quality and are discussed accordingly, highlighting the need for care to be safe, effective, caring, responsive and personalized, and for healthcare organizations and systems to be well-led, sustainably resourced, and equitable (27).

More specifically, our findings elucidate the need to deliver safer and more effective care by providing services informed by consistent and up-to-date information and protocols, adequately training professionals, and establishing effective public health measures. In particular, the need for efficient, effective and easy-to-implement protocols was highlighted across settings, together with the need for more responsive and personalized person-and-family-centered care based on shared-decision making, illustrating the importance of developing adaptive expertise within our systems of care. Healthcare protocolization and standardization can aid in ensuring that patients receive evidence-based care and that medical errors are kept to a minimum. Protocols and guidelines, however, are not foolproof solutions to medical decision-making, and clinicians are increasingly faced with new challenges that cannot be met with standard solutions (28). Promoting the development of adaptive expertise among healthcare professionals will allow them to engage in innovative problem-solving, as needed, to meet their patients’ and their families’ specific needs. This can lead to improved patient outcomes, as well as more efficient and effective use of healthcare resources. Finally, the need for effective public health measures to keep citizens well-informed, slow the spread of the virus, and protect and promote mental and physical health was also identified by both patients and professionals. Although strict regulations helped decrease community transmission, they also had significant health, economic, social and psychological consequences, such as decreased access to non-COVID-19 care, job loss, disruptions to education, and increased mental health problems, that must be considered in the future to minimize the overall negative impact on the well-being of the population (29–32).

Further, the need to deliver more caring services embedded within a culture of respect, in which people are treated with compassion and dignity, and that places value on active listening, effective communication, and building trusting relationships was identified. Similarly, the importance of taking care of healthcare professionals’ physical and emotional health also emerged, which was implications for professionals and patients as previous research indicates the emotional healthcare culture impacts patient safety and quality patient care (33).

Moreover, results showed the need for organizations and systems to be (i) well-led, driven by real support from leaders and managers and recognition of the work of professionals; (ii) sustainably resourced, focused on identifying and addressing gaps in human and material resources to meet the rise in demand for care and avoid the saturation of health systems, and (iii) equitable, to reduce inequalities in vulnerable populations such as nursing infants, people in low-income households, older adults, disabled people, and terminally-ill people, and to ensure access to high-quality care and outcomes for all. The COVID-19 pandemic has revealed the importance of seamless collaboration between health and social systems of care. The virus has put a strain on both systems, with healthcare facilities overwhelmed by the number of patients requiring treatment and social systems struggling to support those affected by the pandemic. In order to effectively manage the pandemic and provide optimal care for patients, it is crucial for health and social care systems to work together smoothly and in concert. This includes sharing information and resources, coordinating efforts, and ensuring that patients receive comprehensive care. In Spain, the necessity to improve coordination between services has been widely recognized (34, 35). Recent work (36) has advocated for a new approach to care that establishes a coordinated, comprehensive, person-and-family-centered social and health care model, that provides rehabilitation services, long-term care, and palliative care, and includes a health and social services catalog managed by professionals, joint protocols of action, referral procedures between sectors, and discharge planning strategies to ensure continuity of care in the home environment. Also, a coordinated, multidisciplinary approach to care is essential for the complex and multifactorial impairments associated with the post-acute sequelae of the SARS-CoV-2 infection that affects various aspects of physical, cognitive, and mental health (37). Ultimately, the quality of care delivered depends to a high degree on a well-functioning network and good intra- and interprofessional collaboration and coordination. The identified needs in this study highlight these very issues in the current healthcare system.

### 4.1. Association between identified needs and effective strategies

The following section discusses the association between the needs and effective strategies identified. To note, although participants were asked to report both COVID-19 healthcare and social-related needs and effective strategies, they were not explicitly instructed to match them. Moreover, even though the surveys were open from March 23, 2021 to July 2, 2021, they gathered perspectives regarding needs and strategies associated with waves one through four of the pandemic. At least partly because of this, some of the identified needs were also reported as effective strategies and some have already been addressed in a large-scale, while some of the reported effective strategies continue to hold the potential to address some of the identified needs and to enhance health emergency preparedness. In addition to the date of survey completion, diversity in responses may also be related to individual needs and preferences, as well as variations in healthcare centers and regions, reflecting the dynamic and evolving nature of the COVID-19 pandemic in different contexts and over time.

Next, we illustrate how some of the reported strategies may address some of the identified needs related to patient-monitoring and follow-up care (Clinical care); psychological health and individualized care (Person-and-family centered care); occupational health and safety, and social support and work-life balance (Caring for the healthcare professional); information and public health disease prevention measures (Protocolization, information, health campaigns and education); infrastructure (Resource availability); and coordination between different services (Organizational strategies/needs).

Related to Clinical care, *establishing rigorous and frequent patient monitoring and follow-up care, with the physician managing the case*, could effectively address several of the identified needs. This approach would potentially enable improvements in monitoring for patients with a history of probable COVID-19 and enhance monitoring after the acute phase of the disease, ensuring comprehensive care throughout the recovery process. In fact, continuity of care, or repeated contact between an individual patient and a doctor, is associated with increased adherence to treatment, reduced hospital use, and greater patient satisfaction (38–40). Moreover, this approach would facilitate the implementation of control measures to verify understanding and adherence to guidelines for proper isolation, which would promote public health and minimize the risk of transmission.

Furthermore, *creating community support networks* can address various unmet patient needs related to Person-and-family centered care. They have been shown to help communities reduce the psychological impact related to infectious disease outbreaks (41). By addressing health from a social perspective, these networks promote holistic well-being, combat loneliness, and prioritize the needs of vulnerable populations. Specific to the COVID-19 pandemic, these networks could offer emotional and practical support for patients and their relatives during home isolation and hospitalization, and provide information and strategies to caregivers. Similarly, *implementing a liaison mental health program* to support individuals impacted by COVID-19, including patients and family members, can provide essential psychological support in the short, medium, and long term (42). This type of programs could offer coping strategies, resources, and comprehensive care for all affected individuals. An additional effective strategy to enhance person-and-family centered care involves the *provision of individualized care via comprehensive care plans*. This approach could facilitate appropriate discharge planning based on symptoms and limitations, as well as provide support in scheduling rehabilitation and other medical appointments following hospitalization. By implementing such plans, patients and their families could receive personalized care and support throughout the recovery process, promoting a more holistic approach to healthcare.

For healthcare professionals, *community support networks* can also play a crucial role in mitigating the impact of the pandemic on their mental health and in facilitating the implementation of measures for effective family reconciliation. These networks could provide support for child care, and also afford professionals the opportunity to engage in activities unrelated to COVID-19, allowing them much-needed respite. Additionally, *implementing specific liaison mental health programs for healthcare workers* has also been shown to support their mental health, and can equip them with strategies for coping with the fear of infection, continuous isolation due to working closely with COVID-19 patients, increased workload, redeployment, and managing stress, anxiety, and depression in a rapidly changing landscape (42, 43). Further, mental health programs could facilitate addressing the identified need for continuous proactive evaluation of healthcare providers’ physical and psychological health, and enable organizations to take tangible actions to address chronic burnout, post-traumatic stress, and other complications. *Splitting teams into morning and afternoon shifts,* for example, was reported as an effective strategy to support the well-being of healthcare professionals, by promoting essential rest time, effective workload management, and reduced working hours. Other tangible actions to support healthcare workers’ mental health include expanding basic need resources and services (e.g., child care, food, alternative housing options), recharge/rest spaces, and additional training programs in the workplace (44).

In relation to information and public health disease prevention measures, *contact tracing teams* have emerged as effective strategies according to home-isolated patients and primary care professionals. These teams could play a crucial role in ensuring correct symptom identification, providing information on the disease, protection measures, and isolation guidelines. Additionally, *awareness campaigns that promote inclusive messages and avoid stigmatizing messages*, were also reported as an effective public health strategy that could effectively address several identified needs such as, reducing discrimination in the workplace upon returning from a COVID-19 leave and preventing the spread of fear through the media. By fostering understanding and empathy while promoting health recommendations, these campaigns could help create a supportive environment, dispel misinformation, and mitigate the spread of COVID-19 and negative social consequences.

Moreover, participants reported *increasing the number of ICU and internal medicine beds with temporary structures* as an effective strategy related to Resource availability. This strategy would enable the provision of proper care by increasing surge capacity. Doing so would facilitate the separation of COVID-19 and non-COVID-19 patients, ensuring the continuation of non-COVID-19 care and minimizing the risk of transmission within healthcare facilities (45).

Lastly, *increased coordination between different healthcare services* was highlighted as an effective organizational strategy for enhancing follow-up care. Enhancing coordination could help avoid duplication, ensure necessary controls are in place, improve communication between healthcare professionals, and facilitate easy access to medical records. Ultimately, these measures would contribute to improvements in the quality of patient care and a more streamlined healthcare system.

### 4.2. Strengths and limitations

A key strength of this study lies in the use of a rich data source on COVID-19 health and social -related needs based on the perspectives of patients and professionals. In addition to including first-person perspectives in the surveys, patients were involved in the survey design process and in revising the final lists of identified patient needs. Finally, our work captures the needs and effective strategies associated with the first four waves of COVID-19, from its inception to July 2021. This provides the opportunity to identify needs and strategies that may help prepare health systems for future public health emergencies, as well as more current needs that may need to be addressed or strategies that need to be more widely implemented to improve the quality of care today.

Our study, however, is not without limitations. First, the depth of the open-ended responses obtained was limited by the cross-sectional survey design. Nonetheless, the design was selected as participation in the study required minimal time commitment (10–20 min at one time point only), while allowing us to identify a broad set of COVID-19 healthcare and social-related needs based on the first-person perspectives of patients and professionals in Spain. Second, our study was not designed to detect changes in needs over time; in fact, some of the needs reported by the participants were also reported as effective strategies and others have already been addressed, such as access to and appropriate use of PPE (46). Third, although we identified a plethora of needs and strategies, we did not identify the level of importance associated with each of them. Finally, although our survey response rate of 41.8% falls within the documented average survey response rate of 35–53% (47), we had difficulties efficiently recruiting participants from all Spanish regions (seven of the 19 Spanish regions were not represented), from a wide range of disciplines (profession was mainly limited to nurses and physicians), and participants younger than 27 and older than 69 years, thereby limiting the generalizability of our findings. Nevertheless, surveys were closed based on thematic saturation.

### 4.3. Future research

Moving forward, it will be important to identify improvement priorities, taking into consideration all stakeholders’ views. To this end, the 327 needs identified during this study will form the basis for a follow-up quantitative study focused on prioritizing and obtaining consensus over the identified needs, identifying the resources needed to address these needs, and estimating the associated costs. Additionally, future work should also aim to include other stakeholders’ perspectives, such as family members and policymakers; assess differences in needs by region; and assess which of the identified needs persist, which have been addressed, and which novel needs have emerged. Finally, improvement recommendations for healthcare organizations and systems using an integrated whole-system approach to ensure sustained improvements are warranted (27).

### 4.4. Implications for practice and policy

Our findings have important implications for future clinical practice and policy (See Table 5 for a summary of recommendations). Altogether, we would recommend thoughtful and deliberate consideration of healthcare professionals’ and patients’ perspectives as best practice in organizational decision-making and policymaking, particularly for global crises of such magnitude. Henceforth, organizations and policymakers should actively integrate previously identified healthcare professionals’ and patients’ needs when choosing and implementing their COVID-19 policy packages. Addressing these needs is an essential intervention to mitigate the effects of the current and future public health emergencies. The COVID-19 pandemic has presented unprecedented needs and challenges that require well-co-coordinated responses across governments, healthcare services, and non-government sectors that consider both the possible benefits and harms, to find a balance between reducing the socioeconomic impact and protecting the physical and mental health of all sectors of the population.

**Table 5:** Summary of recommendations for practice and policy.

Theme	Recommendations
Accessibility	Ensure equitable distribution of resources, particularly in under-resourced communities, to reduce disparities in access to COVID-related diagnosis and care. Provide accessible resources, such as translated materials and interpretation services, to help people understand symptoms and take appropriate action. Ensure that all communication is accessible, including adapted materials. Expand access to diagnostic testing, including mobile testing units, drive-through testing centers, and partnerships with community organizations. Ensure emergency care facilities are sufficient and well-equipped for everyone, including people with disabilities. Develop clear guidelines and protocols for the delivery of non-COVID health services during the COVID-19 pandemic.
Basic needs	Implement programs to support the delivery of essential supplies directly to individuals in isolation, such as medication and meal delivery services. Ensure that social and healthcare services collaborate to offer low-income people and families financial aid so they can access basic necessities and support services. Ensure patients’ basic needs are met while hospitalized, including access to showers and healthy nourishing meals. Implement evidence-based design principles to promote healing environments, such as incorporating nature, reducing noise levels, and improving lighting. Provide training and resources to care providers on creating healing environments and reducing environmental stressors.
Clinical care	Increase funding for COVID-19 research and treatment to ensure that patients receive effective treatments. Conduct regular audits and evaluations of COVID-19 and non-COVID-19 care to identify areas for improvement and ensure high-quality care. Ensure that patients and families have access to up-to-date information on COVID-19 treatments, medications, and side effects. Develop comprehensive discharge planning processes to ensure continuity of care, including psychological support. Implement a system for tracking and monitoring patient progress and compliance with follow-up care to ensure continuity of care.
Person-and-family centered care	Develop and implement plans that prioritize the needs and perspectives of patients and families. Provide comprehensive training for healthcare providers on effective communication, empathy, and cultural sensitivity. Foster a culture of respect and promote open and honest communication and collaboration between patients, families, and providers. Focus on addressing the emotional and psychological needs of patients and families by integrating mental health into the overall care pathway. Provide mental health resources and offer counseling and support groups for patients and families affected by COVID-19.
Caring for the healthcare professional	Develop and implement a comprehensive occupational health and safety program for healthcare professionals, including measures to reduce exposure to COVID-19 and support their mental health. Ensure access to mental health services and support groups for healthcare workers. Promote and recognize the value of self-care for healthcare workers, including encouraging breaks and time off work. Provide financial and administrative support to healthcare workers, including hazard pay and coverage for work-related illnesses. Foster a culture of support among healthcare leaders and managers, promoting open dialogue and addressing the needs of healthcare professionals in a timely and empathetic way.
Protocolization, information, health campaigns, & education	Develop and implement clear, evidence-based protocols for the management of COVID-19 patients, taking into account the latest scientific findings and best practices. Provide regular training and education for healthcare professionals on the latest protocols and guidelines for COVID-19 care. Develop adaptive expertise to allow for individualized evidence-based care that takes into account patients’ unique context and needs. Establish a centralized repository for COVID-19-related information and guidance, including regular updates on best practices and new developments.
Resource availability	Increase investment in healthcare infrastructure, including digital and non-digital resources, personnel, and essential materials, to meet the demands of the COVID-19 pandemic. Maximize resource utilization and minimize waste by fostering partnerships and collaborations between government, the private sector, and community organizations. Develop and implement contingency plans to ensure resource availability in the event of future outbreaks or emergencies.
Organizational needs/strategies	Establish clear lines of communication and set up regular check-ins and meetings to ensure all members in the organization are well-informed. Provide support for job retraining and skill development to help workers transition to new roles. Offer flexible work arrangements and paid leave for employees who may need to quarantine or care for significant others. Foster an environment where employees feel comfortable sharing new ideas and suggestions. Enable adaptive capacity to improve the quality of care provided. Ensure that the organization has the resources and technology to maintain services and support employees in times of crisis.

## 5. Conclusion

Here we identify key themes and subthemes related to healthcare and social-related needs and effective strategies associated with COVID-19 from the point of view of patients and professionals in Spanish hospital and primary care settings. Results support the need to provide care that is accessible, high-quality, person-and-family centered, caring, and well-informed, and for organizations and systems to be well-equipped, well-led, and with adapting capacity, to improve the physical and mental health of all. This work will inform healthcare leaders, managers and policy-makers about the main needs and effective strategies perceived by patients and professionals to enable better future preparedness for resilience and contingency planning by administrations and organizations at different levels of care.

## Data availability statement

The raw data supporting the conclusions of this article will be made available by the authors, without undue reservation.

## Ethics statement

This study was reviewed and approved by Hospital Universitario 12 de Octubre Research Ethics Committee (No. 20/608). All participants provided electronic informed consent to participate in this study.

## Author contributions

AD-D, MAB, CO, LP-P, and AG-G: conceptualization and methodology. AD-D and MAB: data curation. AD-D, MAB, and CB-C: formal analysis. AG-G: funding acquisition. AD-D, MAB, CO, LP-P, JB-C, MB-M, CB-C, and AG-G: investigation. MAB and CO: project administration. CO, LP-P, and AG-G: resources and supervision. AD-D, MAB, and LS: visualization. AD-D, MAB, LS, JB-C, and MB-M: writing – original draft. AD-D, MAB, LS, CO, LP-P, JB-C, MB-M, CB-C, and AG-G: writing – review and editing. All authors revised drafts of the manuscript and approved the final version.

## Funding

This work was funded by the Foundation for Biosanitary Research and Innovation in Primary Care (FIIBAP) and the Regional Ministry of Health of the Community of Madrid through non-refundable grants from the credits awarded to the Community of Madrid by the Spanish Government Fund COVID-19, included in Order HAC/667/2020.

## Conflict of interest

The authors declare that the research was conducted in the absence of any commercial or financial relationships that could be construed as a potential conflict of interest.

## Publisher’s note

All claims expressed in this article are solely those of the authors and do not necessarily represent those of their affiliated organizations, or those of the publisher, the editors and the reviewers. Any product that may be evaluated in this article, or claim that may be made by its manufacturer, is not guaranteed or endorsed by the publisher.
