# Supplementary material for: COVID-19 healthcare and social-related needs from the perspective of Spanish patients and healthcare providers: a qualitative analysis of responses to open-ended questions
Source: Front Public Health. 2023 Sep 14;11:1166317. doi: 10.3389/fpubh.2023.1166317 (PMC10538718; doi:10.3389/fpubh.2023.1166317)
Supplement: Supplementary file 1 [file Table_1.docx]

Supplementary Material 1

COVID-19 healthcare and social-related needs from the perspective of Spanish patients and healthcare providers: A qualitative analysis of responses to open-ended questions

Andrea Duarte-Díaz ^†^, Mariana Aparicio Betancourt ^*,†^, Laura Seils, Carola Orrego, Lilisbeth Perestelo-Pérez, Jaime Barrio-Cortes, María Teresa Beca-Martínez, Carlos Jesús Bermejo-Caja, Ana Isabel González-González

† These authors contributed equally to this work and share first authorship.

*** Correspondence:** Mariana Aparicio Betancourt: maparicio@fadq.org

# Supplementary Material 1. Spanish to English translated survey questionnaires assessing COVID-19 healthcare and social-related needs from the perspective of Spanish patients and healthcare providers

**Contents**

[1 S1.1. Survey for people who were home-isolated with COVID-19 3](#_Toc134607629)

[2 S1.2. Survey for people who were hospitalized with COVID-19 8](#_Toc134607630)

[3 S1.3. Survey for hospital care professionals 14](#_Toc134607631)

[4 S1.4. Survey for primary care professionals 19](#_Toc134607632)

# S1.1. Survey for people who were home-isolated with COVID-19

**INFORMED CONSENT**

We are conducting the study " Healthcare and social-related needs identified during the care pathway for people with a history of COVID-19 in Spain: A cross-sectional mixed-methods study".

The aim of this study is to detect the healthcare and social-related needs based on the experience of people with a history of COVID-19and the professionals involved in their care.

The research team is comprised of the following institutions: The Avedis Donabedian Foundation (FAD), the Primary Care Management (GAAP) of the Madrid Health Service, and the Evaluation and Planning Service of the Canary Islands Health Service (SESCS). This project emerged from a collaborative initiative within the framework of the Chronic Diseases Health Services Research Network (REDISSEC) and as a result of the health crisis caused by COVID-19.

Your participation in this study is completely voluntary and you may decide not to participate or to withdraw from the study at any time, without any consequences to your health care.

In order to participate and before proceeding with the survey, please read the project’s information sheet.

You can find more details about the project by accessing our information brochure.

I confirm:

- I have read the information sheet.
- I have been able to ask questions about the study or know how to contact the researchers if I have any questions.
- I have received enough information about the study.

I understand that my participation is voluntary.

I understand that I can withdraw from the study:

1. At any time,
2. Without having to explain myself,
3. Without any consequences on my medical care.

In accordance with the provisions of Regulation (EU) 2016/679 of the European Parliament and of the Council of 27 April on Data Protection (GDPR) and the Organic Law 3/2018 of 5 December on the Protection of Personal Data and the Guarantee of Digital Rights, I declare that I have been informed of my rights, the purpose of collecting my data and the recipients of the information.

If you wish to participate in this study, please check the box "I agree". Clicking "I agree" indicates that you freely agree to participate in the study and consent to the access and use of your data under the conditions detailed in the information sheet. You have the option to save and print a copy of the informed consent form if you wish.

**If you do not wish to participate in this study, you may close this browser tab. Thank you for your time.**

1. Informed consent

- Yes, I agree with the informed consent and agree to answer the questions in this survey
- I do not agree with the informed consent

2. I am of legal age

- Yes
- No

The great demand for human and material resources generated by the COVID-19 pandemic has hindered the response capacity of the National Health System and has required multiple prioritization efforts.

We are interested in identifying the most important improvement areas from the perspective of patients throughout their journey of care to help generate ideas and solutions, and to incorporate them in the planning of resources and development of strategies.

Thus, the objective of this survey is to identify the most important improvement areas in the journey of care for people with a history of COVID-19, from the point of view of people who have had COVID-19 and required home isolation (healthcare professionals who have had COVID-19 and who did not require hospitalization can also participate).

If you have difficulties understanding a question or completing the questionnaire on your computer or cell phone, your family members or caregivers can help you complete the survey as long as the answer represents only your opinion and not that of your family members/caregivers.

If you have any questions or require technical support, please do not hesitate to contact us at fad@fadq.org or at 932076608.

**1. Age**

|  |
| --- |

**2. Gender**

- Man
- Woman
- Non-binary

**3. Date of symptom onset or COVID-19 diagnosis**

|  |
| --- |

**4. Have you fully recovered?**

- Yes
- No

**5. If you have fully recovered, how long have your symptoms lasted (in days)?**

|  |
| --- |

**6. If you have not fully recovered, what symptoms do you continue to have?**

|  |
| --- |

**7. Additional information about any subsequent symptoms or discomfort that you would like to explain to us:**

|  |
| --- |

**8. Spanish region**

|  |
| --- |

**9. Postal code**

|  |
| --- |

**10. Education level**

- No formal education
- Primary education
- Secondary education
- Higher technical education
- University degree or equivalent

**11. Current occupation (If you are currently without an occupation, indicate the number of months without occupation)**

|  |
| --- |

**12. Are you healthcare personnel?**

- Yes
- No

**13. Number of people living in your home**

|  |
| --- |

Next, we ask you a series of questions about the difficulties or aspects to be improved during each moment of your care journey. We are interested in capturing your experiences and perceptions about the main needs identified in any of the phases and waves of COVID-19 in Spain. We would be grateful if you could provide us with details based on your personal experience.

If you feel you are not able to answer a specific question, please state that you do not know this information in the text box.

**1. Contact/symptom onset. What difficulties or improvement areas did you identify when you first contacted the healthcare system suspecting that you might have COVID-19?**

(For example, problems contacting health services, personal discomfort).

|  |
| --- |

**2. Diagnosis: Did you have any problems or did you identify any areas of improvement when you were diagnosed with COVID-19?**

(For example, problems communicating with health services; difficulties with diagnostic testing and obtaining treatment; personal, work and environmental discomfort; difficulties during isolation; monitoring the evolution of the disease).

|  |
| --- |

**3. Visits: What difficulties or aspects to improve did you identify when seen by a healthcare provider?**

(For example, problems in contacting Primary Care services, difficulty in getting to the health center, problems due to isolation in the health center (contact with family members), care and communication with healthcare personnel).

|  |
| --- |

**4. Telephone monitoring and home isolation: What problems or areas of improvement did you identify during home isolation?**

(For example, communication problems with health services; difficulties with diagnostic testing and obtaining treatment; personal, work and environmental discomfort; difficulties during isolation; monitoring the evolution of the disease).

|  |
| --- |

**5. Discharge: What difficulties did you encounter at the time of discharge?**

(For example, work problems, discrimination).

|  |
| --- |

**6. Effective strategies. In your opinion, what aspects of your care were positive in terms of COVID-19 management?**

|  |
| --- |

**We are very grateful for your participation in our survey.**

# S1.2. Survey for people who were hospitalized with COVID-19

**INFORMED CONSENT**

We are conducting the study "Healthcare and social-related needs identified during the care pathway for people with a history of COVID-19 in Spain: A cross-sectional mixed-methods study".

The aim of this study is to detect the healthcare and social-related needs based on the experience of people with a history of COVID-19and the professionals involved in their care.

The research team is comprised of the following institutions: The Avedis Donabedian Foundation (FAD), the Primary Care Management (GAAP) of the Madrid Health Service, and the Evaluation and Planning Service of the Canary Islands Health Service (SESCS). This project emerged from a collaborative initiative within the framework of the Chronic Diseases Health Services Research Network (REDISSEC) and as a result of the health crisis caused by COVID-19.

Your participation in this study is completely voluntary and you may decide not to participate or to withdraw from the study at any time, without any consequences to your health care.

In order to participate and before proceeding with the survey, please read the project’s information sheet.

You can find more details about the project by accessing our information brochure.

I confirm:

- I have read the information sheet.
- I have been able to ask questions about the study or know how to contact the researchers if I have any questions.
- I have received enough information about the study.

I understand that my participation is voluntary.

I understand that I can withdraw from the study:

1. At any time,
2. Without having to explain myself,
3. Without any consequences on my medical care.

In accordance with the provisions of Regulation (EU) 2016/679 of the European Parliament and of the Council of 27 April on Data Protection (GDPR) and the Organic Law 3/2018 of 5 December on the Protection of Personal Data and the Guarantee of Digital Rights, I declare that I have been informed of my rights, the purpose of collecting my data and the recipients of the information.

If you wish to participate in this study, please check the box "I agree". Clicking "I agree" indicates that you freely agree to participate in the study and consent to the access and use of your data under the conditions detailed in the information sheet. You have the option to save and print a copy of the informed consent form if you wish.

**If you do not wish to participate in this study, you may close this browser tab. Thank you for your time.**

1. Informed consent

- Yes, I agree with the informed consent and agree to answer the questions in this survey
- I do not agree with the informed consent

2. I am of legal age

- Yes
- No

The great demand for human and material resources generated by the COVID-19 pandemic has hindered the response capacity of the National Health System and has required multiple prioritization efforts.

We are interested in identifying the most important improvement areas from the perspective of patients throughout their journey of care to help generate ideas and solutions, and to incorporate them in the planning of resources and development of strategies.

Thus, the objective of this survey is to identify the most important improvement areas in the journey of care for people with a history of COVID-19, from the point of view of people with COVID-19 who required hospital admission (healthcare professionals who have had COVID-19 requiring hospitalization can also participate).

If you have difficulties understanding a question or completing the questionnaire on your computer or cell phone, your family members or caregivers can help you complete the survey as long as the answer represents only your opinion and not that of your family members/caregivers.

If you have any questions or require technical support, please do not hesitate to contact us at fad@fadq.org or at 932076608.

**1. Age**

|  |
| --- |

**2. Gender**

- Man
- Woman
- Non-binary

**3. Date of COVID-19 infection**

|  |
| --- |

**4. Date of hospitalization**

|  |
| --- |

**5. Was admission to the ICU required?**

- Yes
- No

**6. Date of hospital discharge**

|  |
| --- |

**7. Have you fully recovered?**

- Yes
- No

**8. If you have fully recovered, how long have your symptoms lasted (in days)?**

|  |
| --- |

**9. If you have not fully recovered, what symptoms do you continue to have?**

|  |
| --- |

**10. Additional information about any subsequent symptoms or discomfort that you would like to explain to us:**

|  |
| --- |

**11. Spanish region**

|  |
| --- |

**12. Postal code**

|  |
| --- |

**13. Education level**

- No formal education
- Primary education
- Secondary education
- Higher technical education
- University degree or equivalent

**14. Current occupation (If you are currently without an occupation, indicate the number of months without occupation)**

|  |
| --- |

**15. Are you healthcare personnel?**

- Yes
- No

Next, we ask you a series of questions about the difficulties or aspects to be improved during each moment of your care journey. We are interested in capturing your experiences and perceptions about the main needs identified in any of the phases and waves of COVID-19 in Spain. We would be grateful if you could provide us with details based on your personal experience.

If you feel you are not able to answer a specific question, please state that you do not know this information in the text box.

**1. Contact/symptom onset. What difficulties or improvement areas did you identify when you first contacted the healthcare system suspecting that you might have COVID-19?**

(For example, problems contacting health services, accessibility problems, personal discomfort, lack of information on isolation measures, lack of information on how to proceed with close contacts, lack of support, etc.).

|  |
| --- |

**2. Diagnosis: Did you have any problems or do you recall any aspects that could be improved during the COVID-19 diagnosis?**

(For example, problems with care and communication with health personnel; accessibility problems; transportation problems; waiting time; lack of information; difficulties with diagnostic testing and obtaining treatment; personal, work and environmental discomfort; difficulty during isolation; monitoring the evolution of the disease; lack of psychological, emotional, and social support).

|  |
| --- |

**3. Emergency care: What difficulties or improvement areas did you identify when seen in an emergency department?**

(For example, problems in contacting emergency services, difficulty in getting to the health center, problems due to isolation in the emergency department (contact with family members), care and communication with healthcare personnel).

|  |
| --- |

**4. Hospitalization: What problems or improvement areas did you identify while you were hospitalized?**

(For example, problems due to isolation in the hospital ward: contact with relatives, care and communication with the healthcare staff with you or your relatives).

|  |
| --- |

**5. ICU care: If admitted to the ICU, what difficulties did you have while being in the ICU? If you do not remember, simply do not answer.**

(For example, problems due to isolation in the ICU, contact with family members, care and communication with healthcare personnel, psychological support).

|  |
| --- |

**6. Hospital discharge. What problems or improvement areas did you identify upon discharge from the hospital?**

(For example, communication problems with health services or health personnel; difficulties in carrying out control tests and obtaining treatment; personal, work and environmental discomfort; difficulties during isolation; monitoring the evolution of the disease; difficulties performing social tasks; long-term physical or mental health problems).

|  |
| --- |

**7. Effective strategies. In your opinion, what aspects of your care were positive in terms of COVID-19 management?**

|  |
| --- |

**We are very grateful for your participation in our survey.**

# S1.3. Survey for hospital care professionals

**INFORMED CONSENT**

We are conducting the study "Healthcare and social-related needs identified during the care pathway for people with a history of COVID-19 in Spain: A cross-sectional mixed-methods study".

The aim of this study is to detect the healthcare and social-related needs based on the experience of people with a history of COVID-19and the professionals involved in their care.

The research team is comprised of the following institutions: The Avedis Donabedian Foundation (FAD), the Primary Care Management (GAAP) of the Madrid Health Service, and the Evaluation and Planning Service of the Canary Islands Health Service (SESCS). This project emerged from a collaborative initiative within the framework of the Chronic Diseases Health Services Research Network (REDISSEC) and as a result of the health crisis caused by COVID-19.

Your participation in this study is completely voluntary and you may decide not to participate or to withdraw from the study at any time without any consequences.

In order to participate and before proceeding with the survey, please read the project’s information sheet.

You can find more details about the project by accessing our information brochure.

I confirm:

- I have read the information sheet.
- I have been able to ask questions about the study or know how to contact the researchers if I have any questions.
- I have received enough information about the study.

I understand that my participation is voluntary.

I understand that I can withdraw from the study:

1. At any time,
2. Without having to explain myself,
3. Without any consequences.

In accordance with the provisions of Regulation (EU) 2016/679 of the European Parliament and of the Council of 27 April on Data Protection (GDPR) and the Organic Law 3/2018 of 5 December on the Protection of Personal Data and the Guarantee of Digital Rights, I declare that I have been informed of my rights, the purpose of collecting my data and the recipients of the information.

If you wish to participate in this study, please check the box "I agree". Clicking "I agree" indicates that you freely agree to participate in the study and consent to the access and use of your data under the conditions detailed in the information sheet. You have the option to save and print a copy of the informed consent form if you wish.

**If you do not wish to participate in this study, you may close this browser tab. Thank you for your time.**

1. Informed consent

- Yes, I agree with the informed consent and agree to answer the questions in this survey
- I do not agree with the informed consent

2. I am of legal age

- Yes
- No

The great demand for human and material resources generated by the COVID-19 pandemic has tested the response capacity of the National Health System and has required multiple prioritization efforts.

The establishment of priorities by the main interest groups (e.g., patients and healthcare providers) and adequate resource planning can facilitate decision-making in relation to health and social policies.

Thus, the aim of this survey is to identify priority improvement areas in the journey of care for people with a history of COVID-19, from the point of view of Hospital Care professionals.

If you have any questions or require technical support, please do not hesitate to contact us at fad@fadq.org or at 932076608.

**1. Age**

|  |
| --- |

**2. Gender**

- Man
- Woman
- Non-binary

**3. Profession**

- Physician
- Nurse
- Other (specify)

|  |
| --- |

**4. Title**

|  |
| --- |

**5. Service**

|  |
| --- |

**6. Spanish region**

|  |
| --- |

**In relation to the care of patients with COVID-19:**

What difficulties, improvement areas, or unmet needs of patients or professionals would you highlight for each of the following stages of hospital care for patients with COVID-19?

We are interested in capturing your experiences and perceptions about the main needs identified in any of the phases and waves of COVID-19 in Spain. We would be grateful if you could provide us with details based on your personal experience.

If you feel that you do not have in-depth knowledge of the area being mentioned, please specify it in the text box.

**1. In the initial emergency care of patients with COVID-19.**

(For example, problems in contacting emergency services, difficulty in getting to the center, problems due to isolation in the emergency department (contact with relatives), attention and communication with healthcare personnel, inability to respond to certain patient needs, etc.).

|  |
| --- |

**2. During hospitalization of patients with COVID-19.**

(For example, problems due to isolation on the hospital ward, contact with relatives, care and communication of the health care staff with you or your relatives).

|  |
| --- |

**3. During admission of COVID-19 patients to ICU.**

(For example, contact with relatives, care and communication with healthcare staff, other organizational or clinical needs).

|  |
| --- |

**4. At the time of hospital discharge of patients with COVID-19.**

(For example, communication problems with health services or health personnel; difficulties in carrying out control tests and obtaining treatment; personal, work, and/or environmental discomfort; difficulties during home-isolation; difficulty monitoring the evolution of the disease; difficulties performing social tasks; long-term physical or mental health problems).

|  |
| --- |

**On a personal level, as a healthcare professional:**

**5. Other aspects of the work environment. What difficulties have you experienced due to workplace changes as a result of the COVID-19 pandemic? What opportunities for improvement would you suggest?**

(For example, no longer seeing other patients, performing tasks that are not in your specialty or discipline, organizational changes, communication between different professionals, frequent changes in action protocols, travel).

|  |
| --- |

**6. Personal and family environment. What difficulties have you encountered on a personal level as a result of the COVID-19 pandemic? What opportunities for improvement would you suggest?**

(For example, isolation, stigmatization, mental health problems).

|  |
| --- |

**7. Effective strategies. In your opinion, what strategies were effective in the management of the COVID-19 crisis in your care setting? (you may mention aspects that have been implemented at the facility, regional, or state level).**

|  |
| --- |

**We are very grateful for your participation in our survey.**

# S1.4. Survey for primary care professionals

**INFORMED CONSENT**

We are conducting the study "Healthcare and social-related needs identified during the care pathway for people with a history of COVID-19 in Spain: A cross-sectional mixed-methods study".

The aim of this study is to detect the healthcare and social-related needs based on the experience of people with a history of COVID-19and the professionals involved in their care.

The research team is comprised of the following institutions: The Avedis Donabedian Foundation (FAD), the Primary Care Management (GAAP) of the Madrid Health Service, and the Evaluation and Planning Service of the Canary Islands Health Service (SESCS). This project emerged from a collaborative initiative within the framework of the Chronic Diseases Health Services Research Network (REDISSEC) and as a result of the health crisis caused by COVID-19.

Your participation in this study is completely voluntary and you may decide not to participate or to withdraw from the study at any time without any consequences.

In order to participate and before proceeding with the survey, please read the project’s information sheet.

You can find more details about the project by accessing our information brochure.

I confirm:

- I have read the information sheet.
- I have been able to ask questions about the study or know how to contact the researchers if I have any questions.
- I have received enough information about the study.

I understand that my participation is voluntary.

I understand that I can withdraw from the study:

1. At any time,
2. Without having to explain myself,
3. Without any consequences.

In accordance with the provisions of Regulation (EU) 2016/679 of the European Parliament and of the Council of 27 April on Data Protection (GDPR) and the Organic Law 3/2018 of 5 December on the Protection of Personal Data and the Guarantee of Digital Rights, I declare that I have been informed of my rights, the purpose of collecting my data and the recipients of the information.

If you wish to participate in this study, please check the box "I agree". Clicking "I agree" indicates that you freely agree to participate in the study and consent to the access and use of your data under the conditions detailed in the information sheet. You have the option to save and print a copy of the informed consent form if you wish.

**If you do not wish to participate in this study, you may close this browser tab. Thank you for your time.**

1. Informed consent

- Yes, I agree with the informed consent and agree to answer the questions in this survey
- I do not agree with the informed consent

2. I am of legal age

- Yes
- No

The great demand for human and material resources generated by the COVID-19 pandemic has tested the response capacity of the National Health System and has required multiple prioritization efforts.

The establishment of priorities by the main interest groups (e.g., patients and healthcare providers) and adequate resource planning can facilitate decision making in relation to health and social policies.

Thus, the aim of this survey is to identify priority improvement areas in the journey of care for people with a history of COVID-19, from the point of view of Primary Care professionals.

If you have any questions or require technical support, please do not hesitate to contact us at fad@fadq.org or at 932076608.

**1. Age**

|  |
| --- |

**2. Gender**

- Man
- Woman
- Non-binary

**3. Profession**

- Physician
- Nurse
- Other (specify)

|  |
| --- |

**4. Title**

|  |
| --- |

**5. Spanish region**

|  |
| --- |

**In relation to the care of patients with COVID-19:**

What difficulties, improvement areas, or unmet needs of patients or professionals would you highlight for each of the following stages of primary care for patients with COVID-19?

We are interested in capturing your experiences and perceptions about the main needs identified in any of the phases and waves of COVID-19 in Spain. We would be grateful if you could provide us with details based on your personal experience.

If you feel that you do not have in-depth knowledge of the area being mentioned, please specify it in the text box.

**1. Contact/Onset of patient symptoms.**

(For example, lack of human and technological means to contact patients, knowledge, standardization of practice, or care algorithms).

|  |
| --- |

**2. Diagnosis.**

(For example, communication problems with laboratories, difficulties in performing diagnostic tests, lack of material resources, diagnostic difficulties during home visits, etc.).

|  |
| --- |

**3. Visits.**

(For example, difficulty commuting to the patient’s home, lack of protective equipment, problems due to isolation, and care and communication with healthcare personnel).

|  |
| --- |

**4. Telephone monitoring and home isolation.**

(For example, communication problems with health services; difficulties in carrying out control tests and obtaining treatment; personal, work and/or environmental discomfort; difficulties during home-isolation; difficulty monitoring the evolution of the disease).

|  |
| --- |

**5. Discharge.**

(For example, problems at work, discrimination).

|  |
| --- |

**On a personal level, as a healthcare professional:**

**6. Work environment. What difficulties have you experienced due to workplace changes as a result of the COVID-19 pandemic?**

(For example, no longer seeing other patients and people with chronic conditions, performing tasks that are not in your specialty, organizational changes, communication between different professionals, frequent changes in action protocols, travel).

|  |
| --- |

**7. Personal and family environment. What difficulties have you encountered on a personal and family level as a result of the COVID-19 pandemic?**

(For example, isolation, stigmatization, physical and mental health impact).

|  |
| --- |

**8. Effective strategies. In your opinion, what strategies were effective in the management of the COVID-19 crisis in your care setting? (you may mention aspects that were implemented at the facility, regional, or state level).**

|  |
| --- |

**We are very grateful for your participation in our survey.**
