# Supplementary material for: COVID-19 healthcare and social-related needs from the perspective of Spanish patients and healthcare providers: a qualitative analysis of responses to open-ended questions
Source: Front Public Health. 2023 Sep 14;11:1166317. doi: 10.3389/fpubh.2023.1166317 (PMC10538718; doi:10.3389/fpubh.2023.1166317)
Supplement: Supplementary file 2 [file Table_2.docx]

Supplementary Material 2

COVID-19 healthcare and social-related needs from the perspective of Spanish patients and healthcare providers: A qualitative analysis of responses to open-ended questions

Andrea Duarte-Díaz ^†^, Mariana Aparicio Betancourt ^*,†^, Laura Seils, Carola Orrego, Lilisbeth Perestelo-Pérez, Jaime Barrio-Cortes, María Teresa Beca-Martínez, Carlos Jesús Bermejo-Caja, Ana Isabel González-González

† These authors contributed equally to this work and share first authorship.

*** Correspondence:** Mariana Aparicio Betancourt: maparicio@fadq.org

# Supplementary Material 2. Spanish to English translated list of identified COVID-19 healthcare and social-related needs from the perspective of Spanish patients and healthcare providers

**Summary of identified needs by themes**

| **Theme** | **No. of needs** | **No. of subthemes** |
| --- | --- | --- |
| [Accessibility needs](#_Theme:_Accessibility_needs) | 58 | 8 |
| [Basic needs](#_Theme:_Basic_needs) | 17 | 3 |
| [Clinical care needs](#_Theme:_Clinical_care) | 30 | 2 |
| [Person-and-family centered care needs](#_Theme:_Person-and-family_centered) | 47 | 4 |
| [Caring for the healthcare professional needs](#_Theme:_Caring_for) | 41 | 4 |
| [Protocolization, information, health campaigns & education needs](#_Theme:_Protocolization,_information) | 67 | 4 |
| [Resource availability needs](#_Theme:_Resource_availability) | 45 | 4 |
| [Organizational needs](#_Theme:_Organizational_needs) | 35 | 3 |
| **Subtotal** (includes needs coded more than once) | **340** | **32** |
| **Total** (excludes needs coded more than once) | **327** | **32** |

# Theme: Accessibility needs (n = 58)

*Subtheme 1: Early symptoms*

| **Identified need** |
| --- |
| *Patients isolated at home* |
| 1. Access to telephone support lines for providing information (e.g., related to close contact, suspected symptoms or illness). |
| 1. Ease of contacting emergency services (112 and 061) and Primary Care at the first signs^†^. |
| *Patients requiring hospital admission* |
| 1. Ease of contacting Primary Care by phone when your symptoms first appear. |
| *Hospital care professionals* |
| 1. Improving access to telephone care for patients at the onset of symptoms. |
| 1. Ability for patients to quickly contact Emergency Services at the onset of symptoms. |
| ^†^ Coded more than once: early symptoms (accessibility needs) and emergency care (accessibility needs). |

*Subtheme 2: Diagnostic testing*

| **Identified need** |
| --- |
| *Patients isolated at home* |
| 1. Quick referral for diagnostic testing from the first signs. |
| 1. Access to diagnostic testing for people experiencing symptoms and close contacts. |
| 1. Quick communication of diagnostic test results. |
| 1. Quick and convenient access to diagnostic testing near one's residence. |
| 1. Ease of contacting Primary Care to receive diagnosis and treatment plan†. |
| *Patients requiring hospital admission* |
| 1. Quick access and referral for PCR and other diagnostic testing from the first symptoms. |
| 1. Access to diagnostic testing for people experiencing symptoms and close contacts. |
| 1. Quick communication of diagnostic test results. |
| 1. Quick and convenient access to diagnostic testing near one's residence. |
| 1. Possibility of diagnosis from Primary Care. |
| 1. Possibility of continuing isolation and carrying out follow-up diagnostic tests from the patient's home. |
| *Hospital care professionals* |
| 1. Use of diagnostic tests for all patients in Emergency Rooms to rule out cases of asymptomatic COVID-19. |
| 1. Quick communication of diagnostic test results. |
| *Primary care professionals* |
| 1. Quick access to effective diagnostic tests that allow for rapid screening from primary care. |
| 1. Quick communication of diagnostic test results. |
| †Coded more than once: diagnostic testing (accessibility needs) and treatment and rehabilitation services (accessibility needs). |

*Subtheme 3: Emergency care*

| **Identified need** |
| --- |
| *Patients isolated at home* |
| 1. Ease of contacting healthcare services in emergency cases, including weekends. |
| 1. Access to ambulance service for emergency transportation. |
| *Patients requiring hospital admission* |
| 1. Ease of contacting emergency services (112 and 061) and Primary Care at the first signs^†^. |
| *Hospital care professionals* |
| 1. Swift access to urgent medical transportation and prompt attention from out-of-hospital emergency services for seamless referral to hospital emergency care. |
| † Coded more than once: early symptoms (accessibility needs) and emergency care (accessibility needs). |

*Subtheme 4: Treatment & rehabilitation services*

| **Identified need** |
| --- |
| *Patients isolated at home* |
| 1. Ease of contacting primary care to receive a diagnosis and treatment plan†. |
| 1. Ease of contact with the professional responsible for your care during home isolation. |
| 1. Ease of contacting the professional responsible for monitoring symptoms. |
| 1. Ease of contacting different hospital specialties. |
| 1. Access to rehabilitation services for addressing potential complications. |
| 1. Access to medium and long-term rehabilitation services. |
| *Patients requiring hospital admission* |
| 1. Ease of communication with healthcare services after discharge. |
| † Coded more than once: diagnostic testing (accessibility needs) and treatment and rehabilitation services (accessibility needs). |

*Subtheme 5: Non-COVID-19 care*

| **Identified need** |
| --- |
| *Patients isolated at home* |
| 1. Access to comprehensive healthcare for patients, addressing all their needs and not just those related to COVID-19. |
| *Patients requiring hospital admission* |
| 1. Ongoing monitoring of individual’s overall health conditions beyond COVID-19 infection. |
| *Hospital care professionals* |
| 1. Access to unrestricted healthcare for COVID-19 patients or patients with respiratory symptoms that require treatment for conditions other than respiratory illness. |
| *Primary care professionals* |
| 1. Continuity of care for other health conditions (chronic, palliative, bedridden, etc.). |

*Subtheme 6: Occupational health services*

| **Identified need** |
| --- |
| *Patients isolated at home* |
| 1. Quick access to the sick-leave request process after diagnosis. |
| 1. Ease of contacting Occupational Health services after diagnosis. |
| 1. Streamlined administrative procedures for sending a sick-leave. |
| 1. Effective communication with Occupational Health services for managing medical clearance. |

*Subtheme 7: Multimedia communication*

| **Identified need** |
| --- |
| *Patients isolated at home* |
| 1. Strategies to facilitate the conduct of online consultations during the presence of symptoms (e.g., user-friendly platforms, provision of step-by-step information through various media). |
| 1. Access to in-person consultations for priority cases of people in isolation. |
| 1. Ease of contacting Primary Care without the necessity of travel. |
| 1. Availability of alternatives beyond telephone care (e.g., video consultations, home visits). |
| *Patients requiring hospital admission* |
| 1. Access to other follow-up alternatives beyond telephone care (e.g., video consultations, home visits) |
| *Hospital care professionals* |
| 1. Possibility of in-home, in-person care with proper prevention measures in place. |
| *Primary care professionals* |
| 1. Availability of alternative options for visits beyond telephone care (e.g., videoconsultations, home visits). |
| 1. Promotion and normalization of telephone consultations in situations where quality service can be effectively delivered. |
| 1. Timely maintenance of in-person visits with essential safety measures in place. |
| 1. Electronic methods for processing sick-leave and avoiding the necessity for patients to travel to the healthcare center. |

*Subtheme 8: Efficient and continuous access*

| **Identified need** |
| --- |
| *Patients isolated at home* |
| 1. Timely access to in-person care (e.g., reducing or eliminating wait times for appointments, reducing or eliminating wait times at the healthcare center, ensuring access to in-person medical care during holidays). |
| 1. Reduction of wait times for Primary Care appointments. |
| 1. Reduction of wait times for appointments with different specialists in the hospital setting. |
| 1. Improved communication and care with healthcare services: quick and continuous access. |
| 1. Establishing priority appointments to ensure timely access to consultations with different primary and hospital care specialists. |
| 1. Administrative solutions to facilitate appointments requests for people displaced in a different autonomous community from their regular place of residence. |
| *Patients requiring hospital admission* |
| 1. Reduction of wait times in Emergency Rooms. |
| *Primary care professionals* |
| 1. Availability of walk-in medical care in emergency cases. |
| 1. Adequate telephone support lines to avoid service saturation. |

#

# Theme: Basic needs (n = 17)

*Subtheme 1: Essential supplies and resources*

| **Identified need** |
| --- |
| *Patients isolated at home* |
| 1. Domestic assistance services during quarantine (e.g., grocery delivery, cleaning, pharmacy, etc.). |
| 1. Facilities for home quarantine and family reconciliation. |
| *Patients requiring hospital admission* |
| 1. Access to an adequate meal service during hospitalization. |
| 1. Support for personal hygiene of hospitalized patients |
| 1. Availability of bathrooms equipped with showers to ensure proper hygiene during hospitalization. |
| *Hospital care professionals* |
| 1. Provision of patients with their personal belongings. |
| *Primary care professionals* |
| 1. Availability of adapted housing that enable the isolation of infected individuals. |
| 1. Complete isolation of infected individuals from those they live with. |
| 1. Support to facilitate proper isolation, particularly for older patients or patients with dependents. |

*Subtheme 2: Work conditions & financial assistance*

| **Identified need** |
| --- |
| *Patients isolated at home* |
| 1. Financial support based on each person's employment situation during home quarantine. |
| 1. Flexibility in working conditions and encouragement of teleworking following discharge. |
| 1. Oportunity for complete recovery prior to receiving medical clearance and returning to work. |

*Subtheme 3: Healing environment*

| **Identified need** |
| --- |
| *Patients requiring hospital admission* |
| 1. Allocation of a private room as soon as possible. |
| 1. Reduction of noise levels in the hospital. |
| 1. Limitation of the number of patients per room/bathroom and availability of well-ventilated rooms†. |
| 1. Avoiding excessive movement of patients to limit contact between different people. |
| 1. Avoiding excessive movement of patients during hospitalization. |
| † Coded more than once: healing environment (basic needs) and Infrastructure (digital & non-digital) (resource availability needs). |

# Theme: Clinical care needs (n = 30)

*Subtheme 1: Improving patient experience*

| **Identified need** |
| --- |
| *Patients isolated at home* |
| 1. Access to effective treatment during home isolation. |
| 1. Improvements in care for people with mild to moderate symptoms. |
| 1. Provision of comprehensive information to patients regarding the recommended treatment plan following a confirmed diagnosis. |
| *Patients requiring hospital admission* |
| 1. Access to effective treatment from the onset of symptoms. |
| 1. Support and information to promote self-care following hospital discharge. |
| 1. Greater reliability of diagnostic tests. |
| 1. Correct interpretation of diagnostic test results. |
| *Hospital care professionals* |
| 1. Provision of written, simple, and accessible information to assist patients and their caregivers in understanding post-discharge care instructions. |
| *Primary care professionals* |
| 1. Improvements in the diagnosis of close contacts, non-household contacts and asymptomatic individuals. |

*Subtheme 2: Patient monitoring & follow-up* *care*

| **Identified need** |
| --- |
| *Patients isolated at home* |
| 1. Implementation of a continuous symptom monitoring unit from the earliest indications. |
| 1. Establishment of rigorous follow-up controls during home isolation. |
| 1. Improvements in monitoring for patients with a suspected history of COVID-19, even in the absence of a confirmed diagnosis through a positive test result. |
| 1. Quick initiation of frequent and rigorous monitoring. |
| 1. Improvements in monitoring after the acute phase of the disease. |
| 1. Frequent telephone monitoring for symptom control (more than once a week). |
| 1. Maintenance of telephone monitoring for the required duration. |
| 1. Rigorous follow-up controls for proper assessment of medical discharge. |
| *Patients requiring hospital admission* |
| 1. Implementation of a continuous symptom monitoring unit from the first signs |
| 1. Frequent telephone follow-up for symptom control. |
| 1. Maintenance of telephone follow-up for as long as necessary. |
| 1. Quick initiation of a rapid, frequent, and rigorous follow-up to monitor and address potential sequelae arising from infection or prolonged hospitalization. |
| 1. Quick access to follow-up consultations after hospital discharge. |
| 1. Continuous follow-up of patients during hospital isolation. |
| *Hospital care professionals* |
| 1. Resources for the proper telephone follow-up of patients after hospital discharge. |
| 1. Facilitating adherence to the guidelines provided at discharge (e.g., through home visits and rigorous clinical follow-up to ensure continuity of care). |
| 1. Ease of performing follow-up tests and obtaining treatments after hospital discharge. |
| *Primary care professionals* |
| 1. Establishment of rigorous follow-up controls during home isolation. |
| 1. Control measures to verify understanding and adherence to guidelines for proper isolation. |
| 1. Strategies for managing the physical impact of isolation. |
| 1. Strategies for managing sleep disruptions. |

# Theme: Person-and-family centered care needs (n = 47)

*Subtheme 1: Culture of respect*

| **Identified need** |
| --- |
| *Patients isolated at home* |
| 1. Empathy, respect, and understanding from healthcare professionals. |
| 1. Being heard by professionals and making decisions together. |
| 1. Transmission of safety and trust by healthcare staff throughout the entire process. |
| 1. Healthcare professionals projecting safety to patients. |
| *Patients requiring hospital admission* |
| 1. Empathy, respect, and understanding from healthcare professionals. |
| 1. Being heard by professionals and making decisions together. |
| 1. Transmission of safety and trust by healthcare staff throughout the entire process. |
| 1. Closer attention in Emergency Rooms, particularly for older people. |
| *Hospital care professionals* |
| 1. Improvements in effective communication between healthcare staff and patients and their families. |
| 1. Closeness in communication between healthcare professionals and hospitalized patients. |
| *Primary care professionals* |
| 1. Humanization of Primary Care. |
| 1. Closer telephone contacts. |

*Subtheme 2: Psychological health*

| **Identified need** |
| --- |
| *Patients isolated at home* |
| 1. Accompaniment during home isolation. |
| 1. Accompaniment and strategies for coping with isolation. |
| 1. Accompaniment and strategies and information for caregivers of patients with COVID-19†. |
| *Patients requiring hospital admission* |
| 1. Psychological support and accompaniment during hospitalization. |
| 1. Psychological support and accompaniment after hospital discharge. |
| *Hospital care professionals* |
| 1. Support and resources to help patients cope with fear and uncertainty, especially in relation to ICU admission. |
| 1. Support and accompaniment for the patient during hospital admission. |
| 1. Strategies for coping with isolation of loved ones. |
| 1. Creation of a mental health liaison program aimed at supporting healthcare workers, patients and family members of people affected by COVID-19¥. |
| 1. Ensuring free access to entertainment options for patients, including television. |
| *Primary care professionals* |
| 1. Addressing health from a social perspective. |
| 1. Support and accompaniment for patients in isolation. |
| 1. Implementation of support, accompaniment and socialization campaigns, following necessary protection measures, for people in need (e.g., older adults, youth, disabled). |
| † Coded more than once: psychological health (person-and-family centered needs) and family involvement (person-and-family centered needs).  ¥ Coded more than once: psychological health (person-and-family centered needs) and psychological health (caring for the healthcare professional needs) |

*Subtheme 3: Individualized care*

| **Identified need** |
| --- |
| *Patients isolated at home* |
| 1. Assigning a healthcare professional as a dedicated case manager for each patient to provide personalized care. |
| 1. Adaptation of recommendations based on specific considerations (e.g., isolation and medication for pregnant women and other vulnerable groups). |
| 1. Clear and conclusive explanation about the reasons for discharge. |
| 1. Medical discharge determined by symptoms and limitations, rather than not solely relying on a temporal criterion. |
| *Patients requiring hospital admission* |
| 1. Access to effective treatment tailored to the individual and their circumstances. |
| 1. Clear and conclusive explanation of the reasons for discharge. |
| 1. Support for scheduling rehabilitation and medical appointments after hospital discharge. |
| 1. Medical discharge determined by symptoms and limitations, rather than not solely relying on a temporal criterion. |

*Subtheme 4: Family involvement and communication*

| **Identified need** |
| --- |
| *Patients isolated at home* |
| 1. Accompaniment and strategies and information for caregivers of patients with COVID-19†. |
| 1. Promotion of the participation of caregivers in follow-up contacts (e.g., participating in calls). |
| *Patients requiring hospital admission* |
| 1. Opportunity for patients to maintain contact with family members and loved ones during their stay in the emergency department.. |
| 1. Possibility of contact with family members and other loved ones (calls, video conferences, etc.) during hospitalization. |
| *Hospital care professionals* |
| 1. Availability of alternatives for communication between hospitalized patients and families. |
| 1. Improvements in effective communication between the patient and their family. |
| 1. Implementation of communication plans to establish ongoing contact with families, facilitating the provision of information and preventing overload of telephone lines.. |
| 1. Daily provision of information for families of patients admitted to ICUs both verbally and in writing. |
| 1. Option for dependent individuals, particularly those requiring assistance, to be accompanied by a trusted person in the emergency department, while ensuring appropriate preventive measures are in place. |
| 1. Possibility of accompaniment of patients by a trusted person with appropriate preventive measures, particularly for older people, people with disabilities, and those in a terminal situation. |
| 1. Possibility of safely maintaining face-to-face communication with family members. |
| 1. Possibility of maintaining contact with the family through different means (telephone calls, video calls), facilitated by the hospital staff if necessary. |
| *Primary care professionals* |
| 1. More frequent communication with the family members of patients isolated at home. |
| 1. Flexibility to facilitate access to consultation for patients with physical or cognitive difficulties, allowing them to be accompanied by a close person throughout the entire care journey. |
| † Coded more than once: psychological health (person-and-family centered needs) and family involvement (person-and-family centered needs). |

# Theme: Caring for the healthcare professional needs (n = 41)

*Subtheme 1: Support from leaders and managers*

| **Identified need** |
| --- |
| *Hospital care professionals* |
| 1. Support and good coordination and leadership from superiors. |
| *Primary care professionals* |
| 1. Real support from management and recognition of the work of professionals. |
| 1. Understanding and support from the management of the Primary Care centers. |
| 1. On-site visits from management to observe day-to-day operations and support professionals. |
| 1. Leadership equipped to provide support to professionals and the organization in effectively adapting to changes brought about by the pandemic†. |
| † Coded more than once: support from leaders and managers (caring for the healthcare professional needs) and organizational changes (organizational needs). |

*Subtheme 2: Occupational health and safety*

| **Identified need** |
| --- |
| *Patients requiring hospital admission* |
| 1. Proper use of personal protective equipment in the Emergency department. |
| *Hospital care professionals* |
| 1. Reinforcement of the importance of hand hygiene and preventive measures among healthcare personnel, promoting their adherence not only during patient care but also during meals, leisure, and rest periods. |
| 1. Thorough COVID-19 prevention measures to avoid infection among healthcare professionals. |
| 1. Need for rest time, both inside and outside the work environment, for healthcare professionals. |
| 1. Access to PPE that keeps healthcare workers cool and does not impede visibility and comfort. |
| 1. Maintenance of an appropriate workload to avoid overworking healthcare professionals. |
| 1. Measures to prevent infection of close contacts. |
| 1. Work demands in accordance with the specific training and expertise of each healthcare professional. |
| *Primary care professionals* |
| 1. Access to PPE that keeps healthcare workers cool and does not hinder visibility and comfort. |
| 1. Extended rest periods for healthcare workers to compensate for overtime. |
| 1. Continuous proactive evaluation of the physical and psychological health of healthcare workers and actions to address chronic burnout and other symptoms†. |
| 1. Option to perform low-risk COVID-19 work for healthcare workers belonging to vulnerable groups. |
| 1. Reduction of workload and working hours for healthcare workers. |
| † Coded more than once: occupational health and safety (caring for the healthcare professional needs) and psychological health (caring for the healthcare professional needs). |

*Subtheme 3: Psychological health*

| **Identified need** |
| --- |
| *Hospital care professionals* |
| 1. Support and accompaniment for the healthcare professional. |
| 1. Attention to the mental health of healthcare professionals. |
| 1. Strategies for coping with the fear of infection (for oneself and close contacts). |
| 1. Creation of a mental health liaison program to help healthcare workers, patients and family members of people affected by COVID-19†. |
| *Primary care professionals* |
| 1. Strategies for managing the fear of infection (of oneself and close contacts). |
| 1. Psychological support and accompaniment for the health professional. |
| 1. Psychological support in managing emotions. |
| 1. Strategies for managing stress and anxiety. |
| 1. Support and accompaniment for coping with the need for continuous isolation as a result of being in permanent contact with COVID-19 patients. |
| 1. Continuous proactive evaluation of the physical and psychological health of healthcare workers and actions to address chronic burnout and other symptoms¥. |
| 1. Permits for outdoor walks following protective measures during confinement. |
| 1. Coping strategies for managing isolation and lack of socialization. |
| † Coded more than once: occupational health and safety (caring for the healthcare professional needs) and psychological health (caring for the healthcare professional needs).  ¥ Coded more than once: occupational health and safety (caring for the healthcare professional needs) and psychological health (caring for the healthcare professional needs). |

*Subtheme 4: Social support & work-life balance*

| **Identified need** |
| --- |
| *Hospital care professionals* |
| 1. Measures for family reconciliation for professionals. |
| 1. Support for child care due to increased working hours. |
| 1. Support for professionals who have contracted COVID-19 (e.g., designated accommodation and food during isolation, care for dependents, etc.). |
| 1. Measures to decrease/prevent stigmatization. |
| 1. Dedicated time to engage in activities unrelated to COVID-19 and reduce the overload of information about COVID-19. |
| *Primary care professionals* |
| 1. Support for professionals in caring for minors and other people under their charge. |
| 1. Strategies to cope with the fear of infection and support for professionals in case of COVID-19 infection. |
| 1. Alternatives to maintain safe contact with loved ones. |
| 1. Measures to decrease/prevent stigmatization of professionals in direct contact with COVID-19 patients. |
| 1. Access to masks for the general public, with a particular focus on providing masks to family members of professionals, to minimize the risk of infection. |
| 1. Implementation of measures for adequate family reconciliation (shift changes, reduced hours, etc.). |
| † Coded more than once: social support & work-life balance (caring for the healthcare professional needs) and general or other material resources (resource availability needs). |

# Theme: Protocolization, information, health campaigns, and education needs (n = 67)

*Subtheme 1: Protocolization*

| **Identified need** |
| --- |
| *Patients isolated at home* |
| 1. Establishing effective, clear, and consistent protocols throughout the entire care process (early symptom identification, diagnosis, treatment, and discharge). |
| 1. Clear protocols for specific situations such as caring for an infant living with caregivers with COVID-19. |
| 1. Specific protocols for the protection and reintegration of particularly vulnerable people to the workforce. |
| *Patients requiring hospital admission* |
| 1. Establishment of effective, clear, and consistent protocols throughout the entire care process (early symptom identification, diagnosis, treatment, and discharge). |
| 1. Clear protocols for rapid referral to emergency care. |
| 1. Establishment of clear protocols for diagnostic tests with inconclusive results. |
| 1. Implementation of effective treatment protocols in emergency departments. |
| *Hospital care professionals* |
| 1. Establishment of effective, clear and consistent protocols throughout the entire care process (early symptom identification, diagnosis, treatment and discharge). |
| 1. Clear and accessible action protocols (e.g., isolation, criteria for ICU admission). |
| 1. Concise and continuous updates on relevant changes to protocols. |
| 1. Implementation of simple and consistent action protocols over time in accordance with existing scientific evidence to avoid unnecessary changes. |
| *Primary care professionals* |
| 1. Establishment of effective, clear and consistent protocols throughout the entire care process (early symptom identification, diagnosis, treatment and discharge). |
| 1. Concise and continuous updates on relevant changes to protocols. |
| 1. Unified and clear action protocols that avoid the need for improvisation by primary care centers, including home care protocols. |
| 1. Clear and consistent action protocols over time. |
| 1. Avoid unnecessary changes in action protocols and care algorithms. |
| 1. Clear protocols in relation to algorithmic interpretation, confinement time and request for tests. |
| 1. Standardization of practice and care algorithms while allowing for a certain degree of flexibility based on clinical judgment and specific circumstances). |

*Subtheme 2: Information*

| **Identified need** |
| --- |
| *Patients isolated at home* |
| 1. Ensuring uniformity in the information provided from different services and professionals (e.g., 012 and Primary Care). |
| 1. Providing information to the public on how to act when symptoms first appear. |
| 1. Information for the correct identification of COVID-19 alarm symptoms that require urgent attention. |
| 1. Provision of information about COVID-19, protective measures and isolation guidelines to patients by health professionals. |
| 1. Health professionals to provide updated, concise and understandable information. |
| 1. Public Health services to provide updated, concise and understandable information. |
| 1. Media to provide updated, concise and understandable information. |
| 1. Updated, concise and accessible information regarding the risk of infection, antibody development and the potential for reinfection. |
| 1. Counseling (including legal advice) for the management of medical discharge and returning to work. |
| 1. Recommendation for immediate isolation of people with suspected infection, probable infection or confirmed infection. |
| *Patients requiring hospital admission* |
| 1. Quick identification of symptoms compatible with the presence of the virus. |
| 1. Ensuring uniformity in the information provided from different services and professionals (e.g., 012 and Primary Care). |
| 1. Health professionals to provide updated, concise and understandable information. |
| 1. Public Health services to provide updated, concise and understandable information. |
| 1. Media to provide updated, concise and understandable information. |
| 1. Provision of sufficient information for hospitalized patients and their families. |
| *Hospital care professionals* |
| 1. Provision of updated and understandable information for patients and families. |
| *Primary care professionals* |
| 1. Provision of updated and understandable information for patients and families. |

*Subtheme 3: Public health disease prevention measures*

| **Identified need** |
| --- |
| *Patients isolated at home* |
| 1. Control measures to ensure strict compliance with isolation protocols. |
| 1. Thorough controls prior to resuming work to prevent the spread of the virus, including PCR testing and medical examinations. |
| 1. Measures to reduce discrimination after returning to work after COVID-19 infection. |
| *Patients requiring hospital admission* |
| 1. Avoiding the spread of fear through the media. |
| *Hospital care professionals* |
| 1. Public education initiatives aimed at increasing understanding of the implications of the disease among the general population. |
| 1. Public health measures to inform the public and avoid hospital saturation. |
| *Primary care professionals* |
| 1. Multimodal education for the public, patients and their close contacts on procedures and measures for infection control (e.g., verbal and written instructions and video tutorials modeling steps to follow). |
| 1. Informative campaigns about the importance of staying isolated from the rest of the people they live with. |
| 1. Maintenance and continuity of primary, secondary and tertiary prevention campaigns. |
| 1. Strategies for managing the fear of non-COVID-19 patients when attending face-to-face visits (e.g., clear information about protective measures). |
| 1. Public health measures to ensure that the public complies with the individual and collective protection measures adapted to the context. |
| 1. Effective contact tracing by Public Health. |

*Subtheme 4: Education & training*

| **Identified need** |
| --- |
| *Patients isolated at home* |
| 1. Early identification of symptoms by health professionals. |
| 1. Increasing the preparation and knowledge about COVID-19 of health professionals. |
| 1. Increasing knowledge and training of health professionals in relation to telephone follow-up of their patients. |
| 1. Increasing knowledge about persistent COVID-19 and the associated complications and sequelae by professionals. |
| *Patients requiring hospital admission* |
| 1. Increase the preparation and knowledge about COVID-19 of healthcare professionals. |
| 1. Increase the preparation and knowledge about the diagnosis of COVID-19 among healthcare professionals. |
| 1. Increase knowledge about COVID-19 among healthcare professionals in charge of follow-up after hospital discharge. |
| *Hospital care professionals* |
| 1. Rigorous and continuous training for healthcare professionals reassigned to a different area or workplace (e.g., treating medical pathologies that are not their specialty). |
| 1. Training and resources for AP professionals in relation to the follow-up of patients with COVID-19 history after hospital discharge. |
| 1. Telehealth training for healthcare professionals. |
| 1. Rigorous and continuous training for healthcare professionals on the rapidly changing COVID-19 situation. |
| 1. Initial training encompassing both theoretical knowledge and practical skills for the management of an unknown condition at that moment. |
| 1. Training and resources for the communication of difficult situations between professionals and patients/families. |
| 1. Maintenance of training activities for residents. |
| 1. Coping strategies for the management of stress and anxiety of patients at the time of discharge. |
| *Primary care professionals* |
| 1. Early identification of symptoms compatible with COVID-19 by healthcare professionals. |
| 1. Rigorous and continuous training for healthcare professionals who are reassigned to different areas or workplaces (e.g., treating medical pathologies that are not their specialty). |
| 1. Training on how to properly use PPE in different environments (e.g., home visits, health centers). |
| 1. Training and resources for primary care professionals in relation to the follow-up of patients with a history of COVID-19 after hospital discharge. |

# Theme: Resource availability needs (n = 45)

*Subtheme 1: Human resources*

| **Identified need** |
| --- |
| *Patients isolated at home* |
| 1. Increase in the number of contact tracers and improvements in logistics for quick contact tracing†. |
| 1. Sufficient administrative personnel at the health center for appointment management. |
| *Patients requiring hospital admission* |
| 1. Availability and hiring of sufficient and qualified human resources to meet the increasing demand for healthcare and prevent service saturation |
| 1. Quick separation of COVID-19 and non-COVID-19 patients^¤^. |
| *Hospital care professionals* |
| 1. Availability and hiring of human resources to meet healthcare demand and avoid service saturation. |
| 1. Hiring of additional staff to avoid having to extend hours, shifts, on-call duty and forced transfers as needed. |
| 1. Sufficient staff to ensure patients can perform activities of daily living (e.g., eating and grooming) during hospitalization. |
| 1. Increase in primary care resources dedicated to screening and initial examination to avoid emergency service saturation¥. |
| 1. Resources for mental health care of the general population and hospitalized patients. |
| 1. Resources to address anxiety and emotional disorders of hospitalized patients and families. |
| 1. Time and resources for healthcare professionals to effectively communicate and report the status of hospitalized patients to their family members through telephone calls. |
| 1. Increase in resources so that healthcare professionals have time to inform families about the patient's health status. |
| 1. Hiring of human resources to avoid abandonment of care for other conditions. |
| 1. Sufficient resources to avoid delays in early diagnostic testing and treatment for non-COVID-19 conditions¥ |
| *Primary care professionals* |
| 1. Availability and hiring of qualified human resources to meet healthcare demand and avoid service saturation. |
| 1. Hiring administrative personnel due to increased demand (contact tracing, IT management, etc.). |
| 1. Hiring social workers to mobilize resources to facilitate the purchase of essential goods for isolated patients. |
| 1. Hiring additional staff for monitoring COVID-19 patients by phone, proactive monitoring of non-COVID-19 patients, and for support with organizational tasks (e.g., triage). |
| 1. Availability of contact tracing teams to monitor contacts when symptoms first appear. |
| 1. Increase in resources for authorities to verify that the infected person complies with the prescribed isolation. |
| 1. Hiring additional staff to avoid the need to redeploy professionals. |
| † Coded more than once: human resources (resource availability needs) and organizational changes (organizational needs).  ¥ Coded more than once: human resources (resource availability needs) and general or other material resources (resource availability needs).  ¤ Coded more than once: human resources (resource availability needs) and infrastructure (resource availability needs). |

*Subtheme 2: Infrastructure (digital & non-digital)*

| **Identified need** |
| --- |
| *Patients requiring hospital admission* |
| 1. Limit the number of patients per room/bathroom and availability of well-ventilated rooms†. |
| 1. Quick separation of COVID-19 and non-COVID-19 patients^¤^ |
| *Hospital care professionals* |
| 1. Ensure sufficient space and proper care for non-COVID-19 treatment in COVID-19 patients. |
| 1. Infrastructures that allow for patient privacy during emergency treatment. |
| 1. Availability of designated spaces for the isolation of confirmed symptomatic positive cases, asymptomatic positive cases, and suspected cases in separate areas to avoid the risk of infection during hospitalization. |
| 1. Availability of sufficient beds in the critical care and internal medicine units. |
| 1. Enabling more ICUs in private and public centers to avoid saturation of services. |
| 1. Availability of specific waiting rooms in emergency departments for COVID-19 patients (positive PCR), possible COVID-19 patients (PCR in process), and non-COVID-19 patients (negative PCR). |
| 1. Implementation of structural changes to effectively avoid crowding in emergency departments. |

| *Primary care professionals* |
| --- |
| 1. Availability of sufficient and quality material resources and infrastructure to meet healthcare demand†. |
| 1. Improvements in technology resources to reinforce digital infrastructure. |
| 1. Telecommunication technology that facilitates good therapeutic relationships between patients and professionals and promotes collaboration among professionals (e.g., phones that use the internet). |
| † Coded more than once: infrastructure (resource availability needs) and general or other material resources (resource availability needs).  ¤ Coded more than once: human resources (resource availability needs) and infrastructure (resource availability needs). |

*Subtheme 3: Transportation*

| **Identified need** |
| --- |
| *Patients requiring hospital admission* |
| 1. Ease of transfer to the patient’s home after hospital discharge. |
| *Hospital care professionals* |
| 1. Availability of ambulances or other suitable means of transportation for patients' transfer to their homes. |
| 1. Facilitate transportation for healthcare workers during COVID-19 confinement (e.g., permits to travel without being stopped by law enforcement checkpoints). |
| 1. Facilitate transportation for healthcare professionals to healthcare centers. |
| *Primary care professionals* |
| 1. Ease and availability of means for healthcare professionals to travel to patients' homes. |

*Subtheme 4: General or other material resources*

| **Identified need** |
| --- |
| *Patients requiring hospital admission* |
| 1. Availability of sufficient material resources to meet the demand for care. |
| *Hospital care professionals* |
| 1. Increase in primary care resources dedicated to screening and initial examination to avoid emergency service saturation¥. |
| 1. Availability of sufficient material resources to meet the demand for care (e.g., reagents). |
| 1. Adequate resources to avoid delays in early diagnostic testing and treatment for non-COVID-19 conditions¥ |
| *Primary care professionals* |
| 1. Availability of sufficient and quality material resources and infrastructure to meet healthcare demand†. |
| 1. Sufficient means for conducting tests such as X-rays or ultrasound at all primary care centers. |
| 1. Access to masks for the general public, with a particular focus on providing masks to family members of professionals, to minimize the risk of infection¤. |
| † Coded more than once: infrastructure (resource availability needs) and general or other material resources (resource availability needs).  ¥ Coded more than once: social support & work-life balance (caring for the healthcare professional needs) and general or other material resources (resource availability needs). |

# Theme: Organizational needs (n = 35)

*Subtheme 1: Coordination and communication*

| **Identified need** |
| --- |
| *Patients isolated at home* |
| 1. Coordination among different services to avoid duplication or lack of controls. |
| *Hospital care professionals* |
| 1. Access to the hospital medical records from Primary Care. |
| 1. Effective communication between hospitals and Primary Care for continuity of care (care of wounds, removal of stitches, check-ups, etc.). |
| 1. Improvements in communication between healthcare professionals. |
| 1. Increased coordination to schedule and communicate the need for redeployment to a different health center to healthcare workers in advance. |
| 1. Facilitating collaboration and consultations between different specialists. |
| *Primary care professionals* |
| 1. Improved communication between Primary Care and the center of reference for proper patient follow-up. |
| 1. Access to the patient's hospital medical history from Primary Care. |
| 1. Coordination between Primary Care and hospital care services (e.g., to avoid duplicity of follow-up after hospital discharge). |
| 1. Coordination and teamwork among Primary Care professionals. |
| 1. Coordination between Primary Care professionals and the contact tracing team upon the appearance of the first symptoms. |
| 1. Improved communication between professionals who request tests and laboratories that perform them. |
| 1. Effective communication between Primary Care, emergency services, public health, and occupational risk prevention. |
| 1. Direct access to the laboratory for both requesting tests and obtaining results. |

*Subtheme 2: Job security, fair pay, and workers’ rights*

| **Identified need** |
| --- |
| *Patients isolated at home* |
| 1. Guarantee workers' rights and promote supporting employees during medical leave. |
| *Hospital care professionals* |
| 1. Ensure job security, fair salaries and continuous payment for health care workers. |
| 1. Honor previously established contracts and agreements with workers and guarantee labor rights. |
| *Primary care professionals* |
| 1. Maintenance of working conditions or agreed changes (workplace, schedules, shifts, tasks, etc.). |
| 1. Financial assistance for employees and self-employed workers on medical leave. |

*Subtheme 3: Organizational changes*

| **Identified need** |
| --- |
| *Patients isolated at home* |
| 1. Increase in the number of contact tracers and improvements in logistics for quick contact tracing†. |
| *Patients requiring hospital admission* |
| 1. Improving the organization of emergency services to prevent preventable errors. |
| *Hospital care professionals* |
| 1. Institutional organization to face future waves and pandemics while ensuring the continuity of other healthcare activities (reducing the need to improvise). |
| 1. Establishment of quality control measures at the organizational level to detect adverse events and implementation of error prevention strategies. |
| 1. Systematic and continuous evaluation of needs and actions to address identified needs in healthcare services. |
| 1. Increased automation of tasks to avoid service saturation. |
| 1. Prioritization of paused activities at the start of the pandemic to promote patient safety. |
| 1. Institutional transparency related to committed errors. |
| *Primary care professionals* |
| 1. Improved organization and functioning of Primary Care to prevent preventable errors in diagnosis (e.g., loss of samples). |
| 1. Possibility of establishing treatment guidelines from Primary Care to prevent hospital overcrowding. |
| 1. Systematic and continuous evaluation of healthcare service needs and implementation of necessary actions to address them. |
| 1. Leadership equipped to support professionals and the organization in adapting to changes as a result of the pandemic¥. |
| 1. Evidence-based objective decision making in collaboration with frontline healthcare professionals representatives. |
| 1. Transparency in decisions made by management and justification for them. |
| 1. Possibility of resuming work functions and activities that were paused due to the pandemic. |
| 1. Access to an updated list of patient phone numbers for proper contact tracing and follow-up. |
| † Coded more than once: human resources (resource availability needs) and organizational changes (organizational needs).  ¥ Coded more than once: support from leaders and managers (caring for the healthcare professional needs) and organizational changes (organizational needs). |
