# Supplementary material for: COVID-19 healthcare and social-related needs from the perspective of Spanish patients and healthcare providers: a qualitative analysis of responses to open-ended questions
Source: Front Public Health. 2023 Sep 14;11:1166317. doi: 10.3389/fpubh.2023.1166317 (PMC10538718; doi:10.3389/fpubh.2023.1166317)
Supplement: Supplementary file 3 [file Table_3.docx]

Supplementary Material 3

COVID-19 healthcare and social-related needs from the perspective of Spanish patients and healthcare providers: A qualitative analysis of responses to open-ended questions

Andrea Duarte-Díaz ^†^, Mariana Aparicio Betancourt ^*,†^, Laura Seils, Carola Orrego, Lilisbeth Perestelo-Pérez, Jaime Barrio-Cortes, María Teresa Beca-Martínez, Carlos Jesús Bermejo-Caja, Ana Isabel González-González

† These authors contributed equally to this work and share first authorship.

*** Correspondence:** Mariana Aparicio Betancourt: maparicio@fadq.org

# Supplementary Material 3. Spanish to English translated list of identified COVID-19 healthcare and social-related effective strategies & positive aspects from the perspective of Spanish patients and healthcare providers

**Summary of effective strategies and positive aspects by themes**

| **Theme** | **No. of effective strategies & positive aspects** | **No. of subthemes** |
| --- | --- | --- |
| [Accessibility](#_Theme:_Accessibility_(n) | 8 | 4 |
| [Clinical care](#_Theme:_Clinical_care) | 10 | 2 |
| [Person-and-family centered care](#_Theme:_Person-and-family_centered) | 8 | 3 |
| [Caring for the healthcare professional](#_Theme:_Caring_for) | 9 | 3 |
| [Protocolization, information, health campaigns, & education](#_Theme:_Protocolization,_information) | 24 | 4 |
| [Resource availability](#_Theme:_Resource_availability) | 6 | 3 |
| [Organizational strategies](#_Theme:_Organizational_needs) | 21 | 2 |
| **Total** | **86** | **21** |

# Theme: Accessibility (n = 8)

*Subtheme: Early symptoms*

| **Effective strategy or positive aspect reported** |
| --- |
| *Patients isolated at home* |
| 1. Early detection through epidemiology and occupational health services. |

*Subtheme: Diagnostic testing*

| **Effective strategy or positive aspect reported** |
| --- |
| *Patients isolated at home* |
| 1. Facilitating access to follow-up PCR tests to take before ending isolation. |
| 1. Expediting access to diagnostic tests. |
| 1. Implementing measures for rapid diagnosis. |
| *Primary care professionals* |
| 1. Establishing accessible active infection diagnostic tests (AIDTs) for early detection. |

*Subtheme: Treatment & rehabilitation services*

| **Effective strategy or positive aspect reported** |
| --- |
| *Patients isolated at home* |
| 1. Providing early treatment of symptoms to avoid complications. |
| 1. Ensuring timely access to rehabilitation services to address and manage potential physical and cognitive sequelae. |

*Subtheme: Efficient and continuous access*

| **Effective strategy or positive aspect reported** |
| --- |
| *Patients isolated at home* |
| 1. Providing quick and effective access to telephone consultations. |

# Theme: Clinical care (n = 10)

*Subtheme: Improving patient experience*

| **Effective strategy or positive aspect reported** |
| --- |
| *Patients isolated at home* |
| 1. Providing effective treatment. |
| 1. Implementing strategies to improve patient experience and satisfaction. |
| *Patients requiring hospital admission* |
| 1. Implementing strategies to improve patient experience and satisfaction. |
| 1. Ensuring adequate and prompt treatment for COVID-19 infection. |
| 1. Increased continued effort of health personnel. |
| 1. Improving quality of care during emergency room admission. |
| 1. Improving quality of care during hospitalization. |

*Subtheme: Patient monitoring & follow-up care*

| **Effective strategy or positive aspect reported** |
| --- |
| *Patients isolated at home* |
| 1. Establishing rigorous and frequent patient monitoring and follow-up care, with physician managing the case. |
| 1. Enhancing ease of contact with healthcare professionals during patient monitoring and follow-up care. |
| *Patients requiring hospital admission* |
| 1. Implementing follow-up care by Primary Care after hospital discharge. |

# Theme: Person-and-family centered care (n = 8)

*Subtheme: Culture of respect*

| **Effective strategy or positive aspect reported** |
| --- |
| *Patients isolated at home* |
| 1. Providing empathetic and kind treatment by professionals. |
| *Patients requiring hospital admission* |
| 1. Providing empathetic and understanding treatment by health personnel. |

*Subtheme: Psychological health*

| **Effective strategy or positive aspect reported** |
| --- |
| *Patients isolated at home* |
| 1. Establishing community support networks. |
| *Primary care professionals* |
| 1. Leading life as “normal” as possible. |
| *Hospital care professionals* |
| 1. Establishing community support networks. |
| 1. Developing a liaison mental health program to help patients and family members of people affected by COVID-19. |

*Subtheme: Individualized care*

| **Effective strategy or positive aspect reported** |
| --- |
| *Patients isolated at home* |
| 1. Providing individualized treatment. |
| 1. Providing individualized patient monitoring and follow-up care. |

# Theme: Caring for the healthcare professional (n = 9)

*Subtheme: Occupational health and safety*

| **Effective strategy or positive aspect reported** |
| --- |
| *Primary care professionals* |
| 1. Ensuring access to and appropriate use of PPE. |
| 1. Enforcing stringent hygiene measures (e.g., frequent hand washing). |
| 1. Enforcing stringent ventilation measures (e.g., ventilation of doctors’ offices). |
| 1. Implementing comprehensive biosecurity measures for the protection of professionals. |
| *Hospital care professionals* |
| 1. Splitting teams into morning and afternoon shifts to reduce the total number of hours per shift. |
| 1. Implementing and promoting hygiene measures (hand washing, environmental hygiene). |

*Subtheme: Psychological health*

| **Effective strategy or positive aspect reported** |
| --- |
| *Hospital care professionals* |
| 1. Developing a liaison mental health program to help health personnel and their families. |

*Subtheme: Social support & work-life balance*

| **Effective strategy or positive aspect reported** |
| --- |
| *Primary care professionals* |
| 1. Promoting gratitude and recognition for healthcare professionals by society. |
| *Hospital care professionals* |
| 1. Establishing community support for health professionals. |

# Theme: Protocolization, information, health campaigns, and education (n = 24)

*Subtheme: Protocolization*

| **Effective strategy or positive aspect reported** |
| --- |
| *Primary care professionals* |
| 1. Ensuring rapid adaptation of action protocols. |
| *Hospital care professionals* |
| 1. Developing care protocols. |

*Subtheme: Information*

| **Effective strategy or positive aspect reported** |
| --- |
| *Patients isolated at home* |
| 1. Provision of information by the contact tracing team. |
| *Primary care professionals* |
| 1. Providing access to up-to-date information. |
| 1. Enhancing professionals’ knowledge of local care and support services and resources, and sharing this information with patients. |
| 1. Ensuring continuous access to onsite information for people who go to primary care centers throughout the pandemic. |

*Subtheme: Public health disease prevention measures*

| **Effective strategy or positive aspect reported** |
| --- |
| *Patients isolated at home* |
| 1. Ensuring rapid isolation of people with suspected, probable, or confirmed infection, including close contacts of infected people. |
| 1. Implementing a contact tracing service. |
| 1. Promoting the use of mobile contact tracing apps. |
| *Primary care professionals* |
| 1. Performing diagnostic tests outdoors. |
| 1. Limiting maximum occupancy capacity in shared spaces. |
| 1. Ensuring rapid isolation of patients with high suspicion of having SARS-CoV-2 infection. |
| 1. Implementing awareness campaigns that avoid stigmatizing messages. |
| 1. Implementing mass screening initiatives. |
| 1. Implementing mass vaccination of the population, starting with the most vulnerable people. |
| 1. Implementing mass diagnostic testing. |
| 1. Ensuring effective and efficient contact tracing to prevent outbreaks. |
| 1. Implementing biosecurity measures for individual and community protection. |
| 1. Implementing mandatory mask-wearing measures. |
| *Hospital care professionals* |
| 1. Promoting the use of face masks. |
| 1. Implementing social distancing and preventive isolation measures. |

*Subtheme: Education & Training*

| **Effective strategy or positive aspect reported** |
| --- |
| *Patients isolated at home* |
| 1. Investing in COVID-19 research. |
| *Patients requiring hospital admission* |
| 1. Implementing continuous training programs for health personnel. |
| 1. Ensuring knowledge and effective management of persistent COVID-19 symptoms. |

# Theme: Resource availability (n = 6)

*Subtheme: Human resources*

| **Effective strategy or positive aspect reported** |
| --- |
| *Primary care professionals* |
| 1. Hiring support personnel. |
| *Hospital care professionals* |
| 1. Ensuring the availability of sufficient human resources, that were already scarce before the pandemic, to meet the demand for care. |

*Subtheme: Infrastructure (digital & non-digital)*

| **Effective strategy or positive aspect reported** |
| --- |
| *Primary care professionals* |
| 1. Adapting outdoor spaces for patient care or promoting the use of open spaces (e.g., waiting rooms). |
| *Hospital care professionals* |
| 1. Increasing the number of ICU and internal medicine beds with temporary structures to ensure provision of non-COVID-19 care. |
| 1. Building new hospitals. |

*Subtheme: General or other material resources*

| **Effective strategy or positive aspect reported** |
| --- |
| *Hospital care professionals* |
| 1. Ensuring the availability of sufficient material resources, that were already scarce before the pandemic, to meet the demand for care. |

# Theme: Organizational strategies (n = 21)

*Subtheme: Coordination and communication*

| **Effective strategy or positive aspect reported** |
| --- |
| *Patients isolated at home* |
| 1. Improving coordination between different services for adequate follow-up care. |
| 1. Ensuring effective referrals between specialists. |
| *Primary care professionals* |
| 1. Enabling team discussions. |
| 1. Conducting team meetings by videoconference. |
| 1. Developing local collaborations for health promotion. |
| 1. Redistributing roles & responsibilities among employees. |
| 1. Fostering unity between colleagues, across professions and hierarchy. |
| *Hospital care professionals* |
| 1. Ensuring effective communication between care services. |
| 1. Implementing a system for the referral of less severe patients to an appropriate healthcare setting, relieving hospital pressure. |

*Subtheme: Organizational changes*

| **Effective strategy or positive aspect reported** |
| --- |
| *Primary care professionals* |
| 1. Separating clinical spaces within the health center. |
| 1. Permitting changes in appointments to respect social distancing. |
| 1. Enhancing the adaptive capacity of the team. |
| 1. Ensuring the ability to make changes in the organization to better adapt to new needs. |
| 1. Encouraging the use of telephone appointments. |
| 1. Allowing working overtime if necessary. |
| 1. Ensuring timely decision-making and timely implementation of measures. |
| *Hospital care professionals* |
| 1. Implementing pre-entry screening protocols to detect potential COVID-19 cases before they enter a building or facility. |
| 1. Conducting a PCR 72 hours after admission. |
| 1. Establishing closed circuits for COVID-19 patients to prevent them from sharing spaces with non-COVID-19 patients. |
| 1. Establishing independent working groups (COVID-19 teams), e.g., on alternate days, to reduce the risk of COVID-19 transmission. |
| 1. Decreasing the number of face-to-face consultations in cases where telephone consultations are appropriate. |
